# Potentiality of Exosomal Proteins as Novel Cancer Biomarkers for Liquid Biopsy

**DOI:** 10.3389/fimmu.2022.792046

**Published:** 2022-06-09

**Authors:** Chunmiao Hu, Wei Jiang, Mingjin Lv, Shuhao Fan, Yujia Lu, Qingjun Wu, Jiang Pi

**Affiliations:** ^1^ Institute of Laboratory Medicine, Guangdong Provincial Key Laboratory of Medical Molecular Diagnostics, School of Medical Technology, The First Dongguan Affiliated Hospital, Guangdong Medical University, Dongguan, China; ^2^ Department of Cellular Biology, Dakewe, Shenzhen, China

**Keywords:** exosomal proteins, biomarkers, cancer diagnosis, liquid biopsy, exosome

## Abstract

Liquid biopsy has been rapidly developed in recent years due to its advantages of non-invasiveness and real-time sampling in cancer prognosis and diagnosis. Exosomes are nanosized extracellular vesicles secreted by all types of cells and abundantly distributed in all types of body fluid, carrying diverse cargos including proteins, DNA, and RNA, which transmit regulatory signals to recipient cells. Among the cargos, exosomal proteins have always been used as immunoaffinity binding targets for exosome isolation. Increasing evidence about the function of tumor-derived exosomes and their proteins is found to be massively associated with tumor initiation, progression, and metastasis in recent years. Therefore, exosomal proteins and some nucleic acids, such as miRNA, can be used not only as targets for exosome isolation but also as potential diagnostic markers in cancer research, especially for liquid biopsy. This review will discuss the existing protein-based methods for exosome isolation and characterization that are more appropriate for clinical use based on current knowledge of the exosomal biogenesis and function. Additionally, the recent studies for the use of exosomal proteins as cancer biomarkers are also discussed and summarized, which might contribute to the development of exosomal proteins as novel diagnostic tools for liquid biopsy.

## Introduction

Tissue biopsy is widely applied as the gold standard for clinical cancer diagnosis. Nonetheless, tissue sampling becomes a substantial challenge once the tumor is adjacent to major blood vessels, which would make the surgical sampling procedures extremely invasive and painful. In addition, there are also some circumstances in which the small dissected tissues of patients may not be sufficient to represent the pathological profile of the primary tumor (1). Due to the above limitations, liquid biopsy has emerged as the most capable substitute for tissue biopsy due to the advantage of the much more easily accessible samples, such as urine, blood, and cerebrospinal fluid. Multiple sources of tumor-derived substances including circulating tumor cells (CTCs), circulating tumor DNA (ctDNA), and exosomes can be detected from these body fluid samples and can be quickly analyzed (2). Hence, reliable real-time information can be acquired that can help in making a cancer prognosis and monitoring the physiological state of patients with repeated non-invasive sampling (3).

Masked by billions of host cells and vast quantities of free DNA released from normal cells, CTCs and ctDNA are scarcely dispersed in blood. Therefore, it is essential to isolate and enrich CTCs and ctDNA with elaborate techniques that possess high selectivity and sensitivity to carry out clinical analysis (4, 5). However, the enrichment of CTCs remains a big challenge for the application of CTCs, which is restricted by limited cancer surface markers and hindered by a rather limited number of CTCs themselves in the body fluids of cancer patients (6). CtDNA is generally thought to be secreted by necrotic and apoptotic cells. Although its abundance is higher than that of CTCs (7), ctDNA fragments have a very short half-life, even less than 1 h, and are rapidly cleared off (8). Based on these difficulties, the development of exosomes for liquid biopsy has gained increasing attention.

One of the greatest strengths of exosome-based liquid biopsy over CTCs (0–1,000 cells per 7.5 ml of blood) (9) and ctDNA is the larger distributed quantities of exosomes in the body fluids (up to 10^11^ exosomes per ml in the blood) (10). It is not an exaggeration that up to 10% of the circulating exosomes in a cancer patient would be tumor-derived exosomes at the late tumor stage (10, 11). Circulating RNA including mRNA and miRNA is also suggested to be a significant functional mediator in some cancers (8), but the worse stability in the plasma and other intricate causes hinder the clinical use of cell-free RNA (12). On the contrary, another advantage of exosomes as a promising diagnostic marker is that the cargos inside exosomes are well-protected by their lipid bilayer, which makes the intact tumor-derived entities such as RNA, DNA, and proteins carrying the comprehensive oncogenic information to be obtained and identified right after exosome isolation (13). An example of that is the family of phosphorylated proteins, which are usually degraded by the free circulating phosphatase, making them very difficult to be detected in the body fluid. However, these phosphorylated proteins can be well isolated from exosomes and stored stably in the exosomal form for as long as 5 years at −80°C (14). The fact that different cargos are sorted into exosomes as a protective vesicle makes multicomponent analysis feasible, which is conducive to a full-scale knowledge of how exosomes function and what the consequence is. Therefore, the sum of tumor-derived exosomes can be representative of the tumor heterogeneity (15). Moreover, combined research data have shown and confirmed that tumor-derived exosomes and their encapsulated proteins play important roles in cancer progression, which indicates the enormous potentiality for tumor-derived exosomal proteins as novel cancer biomarkers in liquid biopsy (16).

Nucleic acids in exosomes have been intensively investigated as cancer biomarkers. Nonetheless, increasing evidence suggests that proteins of tumor-derived exosomes circulating in the body fluids can also be the precise representative of relevant tumors in distant tissues. Here in this review, the potential of exosomal proteins as liquid biopsy biomarkers in future clinical research has been discussed. The biogenesis and function of exosomes, along with the latest isolation and characterization methods, have been discussed. The application of different exosomal proteins as tumor liquid biopsy targets in different research studies and the future development of exosomal proteins as powerful diagnostic targets in hospitals have been elaborated on in this review.

## Biology and Function of Exosomes

### Biogenesis and Composition of Exosomes

**Figure 1:**
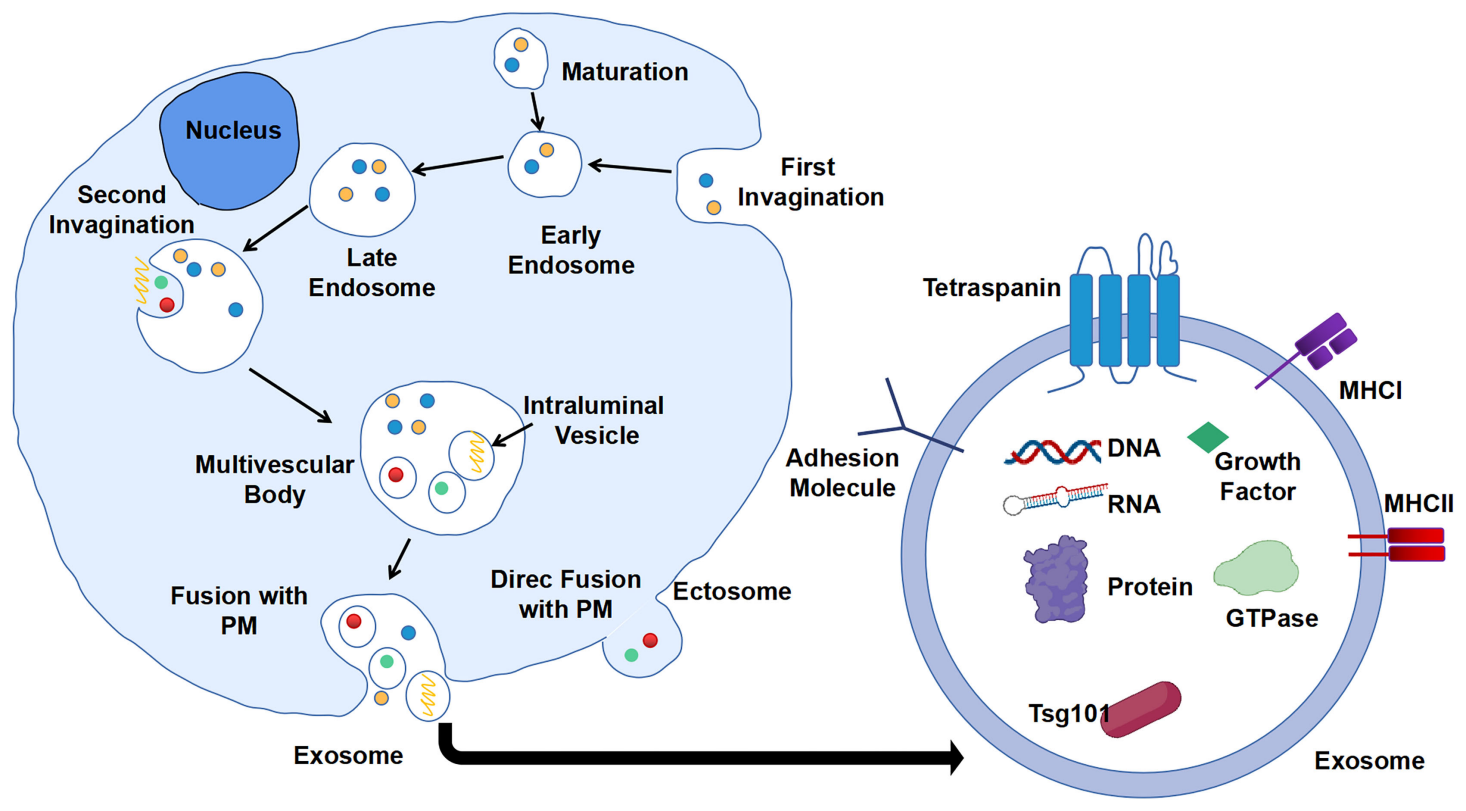
Biogenesis and composition of exosomes. First invagination of plasma membrane (PM) forms the early endosomes that contain multiple constituents, which will then mature into late endosomes. Second, invagination of membrane of late endosomes generates multi-vesicular body (MVB) containing intraluminal vesicles (ILVs). ILVs are secreted into extracellular milieu as exosomes with cargos including DNA, RNA, and proteins.

Extracellular vesicles (EVs) are particles naturally secreted by cells. They are delimited by a lipid bilayer but do not have a functional nucleus for self-replication (17). According to the process of formation, EVs are classified into two categories: ectosomes and exosomes. Ectosomes are formed by direct exocytosis of the plasma membrane (PM) (18). By contrast, exosomes are the product of consecutive internalizations of PM and are finally released by fusion with PM. Consecutive PM internalizations include the first inward budding of PM to form early endosomes, which will then mature into late endosomes. Second, invagination of the membrane in late endosomes gives rise to several intraluminal vesicles (ILVs) containing various constituents such as DNA, RNA, enzymes, and proteins. These late endosome-containing ILVs are also recognized as multi-vesicular body (MVB). Finally, MVB will fuse with PM, and ILVs will be released as exosomes into the extracellular milieu with PM-derived membrane (19, 20) (**Figure 1**). The generation of exosomes can be both ESCRT (endosomal sorting complexes required for transport)-dependent and ESCRT-independent (21).

Exosomes can be secreted by all types of cells including eukaryotic and prokaryotic cells (14, 20). Negatively stained exosomes display classic cup-shape and lipid bilayer structure under electron microscopy (EM), with a generic size range of 40–160 nm (~100 nm on average) in diameter and a density range of 1.15–1.19 g/ml (22). Diverse contents including nucleic acid, protein, lipid, and metabolite can be sorted and packaged into exosomes, which reflect the biological properties of parental cells in different scenarios.

Similar to cell purification and isolation, proteins are widely used as surface and internal markers for the isolation, characterization, and investigation of exosomes. According to the statistical study of data from ExoCarta, a database of exosome cargos, there are approximately 10,000 different exosomal proteins that have been characterized, and the number is believed to be growing over time (23). The proteins wrapped up in exosomes can be divided into three major groups according to their functions: biogenesis relevant (tetraspanins and ESCRT machinery), transport and secretion relevant (heat shock proteins and membrane transport proteins and cytoskeletal proteins), and cell of origin relevant (major histocompatibility complex (MHC) molecules and cytokines and other proteins) (10). CD9, CD63, CD81, and CD82 are the main tetraspanins that are associated with cargo selection machinery and biogenesis of exosomes, and CD63 is the protein marker mostly utilized for immunocapture of exosomes. Representative ESCRT machinery proteins are TSG101 and Alix. Heat shock protein, Rab GTPase, annexin, flotillin, syntaxin, actin, and tubulin are the common exosome transport-related proteins. However, the role of heat shock proteins in the biogenesis of exosomes needs further investigation (24). Some specific exosomes also contain MHC molecules of antigen-presenting cells (APCs) and cytokines like interleukins that are accommodated with immune responses (19).

Exosomal nucleic acids are comprised of DNA, mRNA, miRNA, and lncRNA. As research moves further, the level of miRNA in exosomes was found to be more condensed over other species of nucleic acids from parental cells (25). Compared to other nucleic acids, miRNA is the most widely studied component in exosomes and is found to play a significant role in exosome-mediated cell–cell communication (26). Additionally, differential expression levels of miRNA are vastly discovered in cancer cells by various clinical studies, indicating that miRNA has great potential for cancer diagnosis (27). Nonetheless, there are still difficulties to integrate exosomal miRNA into cancer diagnosis and therapy (28).

In fact, the history of exosome research is relatively short, which is only several decades. The initial discovery of the existence of exosomes was in 1946 (29). But until 1987, Johnstone et al. provided pivotal evidence for the selective release of vesicles that are approximately 50 nm (in diameter) as reticulocytes maturing to erythrocytes. These vesicles were first termed exosomes by the same group (30). Later, a growing body of research data demonstrated in detail that exosomes played important roles in cell–cell communication in both healthy and disease conditions (26, 26, 31, 32), which therefore attracted increasing attention from the scientific field to explore the potential of exosomes as the diagnostic analyte and drug delivery carrier ever since.

### Function of Exosomes

The biogenesis and content of exosomes have determined their destiny and mission as intercellular messengers. When parental cells produce and secrete exosomes, those exosomes carrying proteins, lipids, metabolites, and nucleotides can transfer *via* the extracellular matrix (ECM) and circulation system and to any site of our body. Recipient cells with different varieties can internalize those exosomes by different patterns, such as clathrin-dependent endocytosis (33) or direct fusion with PM (34). Once uptaken, exosomes will deliver their cargos into cells for the regulation of multiple cellular activities, such as cellular development, immune response, and disease condition of recipient cells (19). For example, exosomal DNA can promote the cGAS-STING signaling pathway and pro-inflammatory response (35). Additionally, exosomal miRNA can also show regulatory functions during pregnancy (36). APCs like macrophages or dendritic cells can produce exosomes that carry MHC molecules with antigen peptides. Afterward, T cells are primed and activated when they uptake those exosomes *via* T-cell receptor (TCR)–MHC–peptide recognition (37).

### Tumor-Derived Exosomes and Their Role in Cancer

It is now widely accepted that exosomes also participate in sophisticated cellular interactions between tumor cells and the tumor microenvironment (TME) in every step of cancer development. First, tumor-derived exosomal miRNA modifies gene expression in epithelial cells or fibroblasts, promoting their malignant transformation (19). Meanwhile, other soluble growth factors are also delivered by exosomes to those tumor-associated cells, activating different signaling events like PI3K/AKT pathway (38) or Akt and ERK pathways (39) and leading to the proliferation of recipient cells. Second, tumor-derived exosomes can promote epithelial-to-mesenchymal transition (EMT) and tumor metastasis by activating the resting cancer cells to aggressively metastasize *via* multiple inducible signaling molecules like Notch1 and HIF1α (40, 41). During TME, hypoxic conditions promote tumor cells to express much more exosomal proteins than those outside the niche, which promotes ECM remodeling and angiogenesis (42, 43). In the meantime, tumor-derived exosomes can assist tumor cells to escape from the surveillance of the immune system and develop the capability of chemoresistance, which further promote tumor progression. Some studies have revealed that tumor-derived exosomes can inhibit the cytotoxicity of natural killer cells and cytotoxic T lymphocytes, both of which are indispensable players of the immune mechanism against tumor progression (44, 45). Macrophages and dendritic cells are the pivotal mediators of innate and adaptive immunity, and they are not spared. Tumor-derived exosomes have shown to induce the polarization of immature macrophages into M2 macrophages and display anti-inflammatory activities favoring the ongoing tumor progression (46). Evidence has also shown that maturation of dendritic cells can be suppressed by tumor-derived exosomes, which affects the antigen priming and the activation of antitumor T cells eventually (47). Intrinsically chemoresistant cancer-associated fibroblasts (CAFs) can generate chemoresistant exosomes by sorting relevant molecules into exosomes. Recipient cells including tumor cells in TME will acquire the ability of chemoresistance after the uptake of these chemoresistant exosomes (48). Monitoring exchange of exosomes *in vivo* remains a difficult subject to investigate, which if resolved may shed more light on their tumor-associated function.

## Isolation and Characterization Methods

The characteristics of exosomes are exploited as powerful targets in liquid biopsy and therapy. Nevertheless, the biggest challenge faced for their further application in clinical research and liquid biopsy is the difficulty in obtaining a purified population of exosomes from different sources of samples and accurately characterizing them as authentic exosomes. Although traditional methods like ultracentrifugation remain the gold standard, their unsuitability becomes increasingly obvious due to the requirement of expensive equipment, prolonged working hours, and a large amount of labor (49, 50). On the contrary, new methods like microfluidics take advantage of different traits of exosomes and create opportunities for the transition of exosome research from bench to bedside. The isolation method based on size exclusion or hydrophobic interaction-related mechanism can separate total exosomes from fluid samples. However, the results and yield of purified exosomes among different isolation methods are distinct from one another (51). In addition, there are specific methods that can select subpopulations of exosomes by surface protein markers. In this section, methods based on exosomal proteins will be briefly discussed in terms of suitability for clinical application.

### Isolation Methods

#### Polymer Co-Precipitation

Polycthylene glycol (PEG) is cheap and easy to obtain, and the whole purification process does not require complex equipment, except for low-speed centrifugation. Therefore, many commercial kits have emerged based on the above strengths of PEG co-precipitation, like ExoQuick, Exo-spin, and Total Exosome Isolation. Commercial kits not only save time but also provide relatively standard experimental procedures to eliminate human errors, which therefore are very favorable to clinical labs. Samples of purified exosomes are often contaminated by junk components during isolation (52). When dealing with a serum that contains plenty of lipid proteins and albumin, it is recommended to remove such interference before the use of kits if high purity is required.

#### Affinity-Based Isolation

The membrane of exosomes is enriched with phosphatidylserines, which can bind to a type I transmembrane protein called Tim-4. The binding specificity was developed as a novel method by Nakai et al. to separate phosphatidylserine-rich exosomes (53). Compared to PEG co-precipitation, separation by this method results in fewer contaminants. However, the yield of exosomes might also include other components such as microvesicles that also contain phosphatidylserine. Interestingly, there are two commercial kits that are likely associated with affinity membrane, including exoEasy from QIAGEN and Capturem from TaKaRa.

#### Ultrafiltration

Based on the size and molecular weight, ultrafiltration is used for exosomes enrichment. However, before loading the sample onto an ultrafiltration column, it is usually recommended to perform sequential centrifugation steps to totally remove cells and debris, followed by a one-time filtration step through a 0.45-μm filter to remove large apoptotic bodies (54). Likewise, ultrafiltration is easy to conduct, and it saves both time and labor. But exosomes can remain retained in the filtration membrane during centrifugation, which not only hinders effective separation but also leads to a big loss of yield with insufficient elution (55). Moreover, when it comes to viscous samples like a serum, ultrafiltration shows lesser efficiency. Therefore, the method should be chosen according to the sample of interest to ensure a better separation of exosomes.

#### Size Exclusion Chromatography

Size exclusion chromatography (SEC) is another popular exosome purification method based on the size property of exosomes. It can separate exosomes efficiently from impurities (proteins, lipids, etc.) with well-maintained biological activity. Nonetheless, traditional SEC requires a very long time to run with the existence of contamination in the products (56, 57). But there is a commercial automated instrument from iZON that can be taken into consideration for clinical uses, which is claimed to optimize the running time from hours to minutes.

#### Immunoaffinity Capture

**Figure 2:**
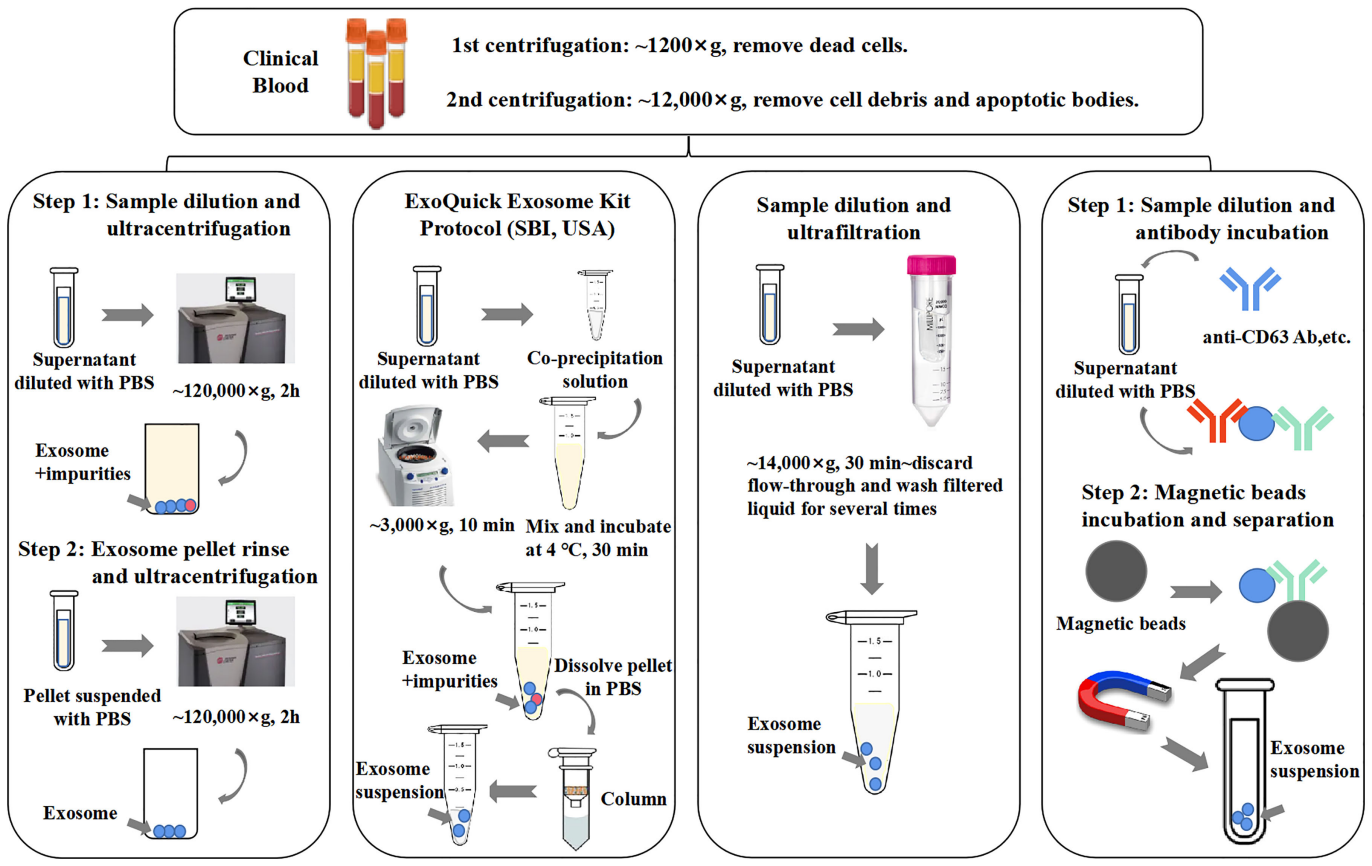
General procedures of different exosome isolation methods. Experimental conditions and operating processes of ultracentrifugation, PEG co-precipitation (ExoQuick Kit, SBI, USA), ultrafiltration, and immunoaffinity are shown in the top panels from left to right. During preparation procedures, blood samples (plasma/serum) would undergo two rounds of low-speed centrifugation, the pellet of each round is deserted, and the supernatant of final round is diluted with phosphate-buffered saline (PBS) for better yield.

Methods of immunoaffinity isolation usually contain two components, streptavidin-coated magnetic beads and biotinylated purified monoclonal antibodies, which can be combined *via* a streptavidin–biotin bond. Antibodies with high affinity will catch corresponding target exosomal proteins to make sure that the exosomes are captured and gathered by a magnet (58). Tetraspanins including CD9, CD63, and CD81 are generally used as targets because these proteins are recognized as ubiquitous markers expressed among exosomes regardless of origin (19). Methods of immunoaffinity do not involve complicated procedures, while the field of monoclonal antibody production is mature enough to guarantee the quality of antibodies. Therefore, the immunoaffinity capture method is appropriate for clinical applications. However, it is worth to be considered that exosomes isolated by the immunoaffinity method cannot get rid of magnetic bead-bound antibodies, which may influence the bioactivity of exosomes and the experimental results of downstream assays. Surprisingly, a new branch of aptamer-based technique emerged recently to show strong potential to resolve the disadvantage of antibody-based technique. The main advantage of the development of aptamers by exosome researchers is that aptamers are easier to synthesize and produce than antibodies (3). Notably, a group successfully separated exosomes with CD63 aptamer and then discharged bound exosomes by adding complementary nucleotide strands to separate exosomes from aptamer. This method could largely maintain the integrity and activity of exosomes, which further reinforces the power of the aptamer-based isolation technique for exosome research (59). **Figure 2** provides an illustrative procedure of representative methods, and **Table 1** provides the comparisons among the mentioned isolation methods with their characteristics.

**Table 1:** Comparison of different exosome isolation techniques.

Isolation technique	Underlying mechanism	Working scalability	Sample purity	Advantage	Disadvantage	Reference
Polymer co-precipitation	Hydrophobicity of protein and lipid	Small	Low	Cheap, fast, easy	Contaminated by co-precipitated particles	(52)
Affinity-based isolation	Affinity	Small	High	Fast, easy	Expensive, contaminated by microvesicles	(53)
Ultrafiltration	Molecular weight	Medium	Medium to high	Fast, easy	Plugged up easily by vesicles	(54, 55)
Size exclusion chromatography	Size and molecular weight	Flexible	High	Cheap, easy, reproducible	Time-consuming	(56, 57)
Immunoaffinity capture	Immunoaffinity-antibody	Small	High	Fast, easy	Expensive, contaminated by magnetic beads	(58)
	Immunoaffinity-aptamer	Small	High	Cheap, fast, easy	Low recovery	(3, 59)
Ultracentrifugation	Differential density	Flexible	Medium	Well-established and commonly used	Time-consuming, ultraspeed centrifuge required	(49, 50)

To achieve a higher yield and purity of exosomes from clinical samples like body fluids, it is recommended to combine different isolation methods along with the pretreatment of clinical samples as mentioned above. In a methodology study of exosome isolation, Lobb et al. showed that ultrafiltration competed over ultracentrifugation, and coupling ultrafiltration with SEC resulted in a high yield of exosomes with high purity (60). They pointed out that the yield of exosomes differed among the application of different isolation methods, which suggested that the size and species of samples need to be taken into account before choosing a compatible isolation method. Another study used ultrafiltration in a company with size exclusion liquid chromatography, which proved that the combination of different methods could also preserve the bioactivity of purified exosomes (61). Scientific and clinical research on exosomes has attracted much attention so far, which impelled the development and revolution of the exosome isolation methodology. Nonetheless, existing methods are still mostly working with tissue cultures, and the results sometimes are not compatible with physical conditions (62). Additionally, the experimental results obtained on similar samples are controversial due to the different amounts of samples and different protocols that are used by different groups (63). Therefore, it is urgent to develop standardized methods to better assist the research of exosomes as well as to facilitate the translation of exosome-based techniques for clinical application in liquid biopsy.

### Characterization Method

#### Microscopy and Nanoparticle Tracking

It is recommended and encouraged to characterize exosomes by two indispensable and complementary techniques, which usually are EM and nanoparticle tracking analysis (NTA) (19). The former provides the image of exosome morphology, and the latter gives information on the size distribution and concentration of exosomes (17). It is necessary to obtain a clear and high-resolution image of exosomes to ensure that the intact structure of exosomes is maintained, which requires hours of microscopy operation and observation (64). Cellular experiments like the uptake of exosomes in the absence of cell–cell contact are sometimes used to demonstrate the bioactivity of purified exosomes. NTA detects the Brownian movement of total nanoparticles of a sample but is unable to distinguish the phenotype or the origin of exosomes (65).

#### Bicinchoninic Acid Assay, Western Blotting, and ELISA

Bicinchoninic acid assay (BCA) is one of the most widely used methods for exosome protein quantification. By combining the examined amount of protein with the concentration of exosome particles, general knowledge of the quantity and purity of the exosome sample can be obtained. Nevertheless, the contaminant proteins can influence the authentic result. Western blotting and ELISA are the most used methods for the characterization of the exosomal composition, and the presence of certain proteins can be confirmed; however, the results are also affected by contaminant proteins, as they cannot separate exosomal proteins from non-exosomal proteins (66). It is suggested by MISEV that at least three positive and one negative protein marker of exosomes should be conducted in Western blotting for exosome characterization (17). For samples like serum or plasma, the most recommended negative markers for exosome characterization are apolipoproteins A1/2 and B as well as albumin (17, 67). However, there are also some contradictory studies indicating the presence of apolipoproteins A and albumin in exosomes (68, 69). Therefore, cytochrome C, a protein of mitochondria, and other endoplasmic reticulum (ER) and Golgi proteins such as calnexin, BIP, GRP94, and GM130 are suggested as negative markers for exosomes instead (17). It is sometimes necessary to remove disturbing proteins to get a cleaner background and a better readout.

#### Flow Cytometric Analysis

Regular flow cytometric devices detect and analyze targets at the cellular level, and thus it is a great challenge to apply them at the nanosized level. To be analyzed by a flow cytometric machine, exosomes should be immobilized on microbeads first and then bound with fluorescent antibodies, which make the fluorescence detectable as a positive signal. The good news is that the recent development of flow cytometric instruments reduces the detection limit to 100 nm (9), which strongly favors future exosome analysis in clinical situations. A major strength of flow cytometry is that it can recognize and select specific subpopulations of exosomes by specific protein markers. Meanwhile, the number of those fluorescence-bearing exosomes can be calculated (70).

#### Microfluidics

As increasing evidence has revealed the significant role of exosomes in cancer progression, some research groups have coined microfluidics for clinical isolation and analysis of exosomes. Usually, microfluidics is a combined system of isolation, characterization, and analysis modules, enabling a complete flow of diagnosis based on exosomes. Microfluidics has the same principles as conventional isolation methods such as immunoaffinity capture and fluorescent analysis. But compared to conventional methods, microfluidics requires much less time and amount of sample, which makes it more clinically favorable. One of the representative examples of microfluidics is developed by the combination with flow cytometry, whose sensitivity reaches 10,000 exosomes per ml of serum (9). We can recognize the microfluidics platform as a scaled-down version of common methods, but with higher sensitivity and throughput, which therefore maintains the great potential for liquid biopsy (72). Nonetheless, the method of microfluidics should be further standardized before being widely applied. A brief comparison of different methods of exosome characterization is summarized in **Table 2**.

**Table 2:** Comparison of different exosome characterization techniques.

Characterization method	Exosome property	Advantage	Disadvantage	Reference
Physical characterization				
Electronic microscopy	Morphology, size distribution	Necessary process for nanosized exosome morphological feature characterization	Time-consuming, affected by human factors like visual sense	(64) (17)
Nanoparticle tracking analysis	Size distribution, concentration in solution	Fast and easy, in combination with microscopy for exosome physical property characterization	Unable to determine the phenotype of exosomes	(17) (65)
Biochemistry characterization				
Western blotting	Presence and level of protein	Classic and standardized method for protein analysis	Low sensitivity, long preparation procedure, unable to exclude contaminants from exosomes	(71) (66)
ELISA	Presence and level of protein	Classic and standardized method for protein analysis	Expensive, unable to exclude contaminants from exosomes	(66)
Flow cytometry	Protein specificity and concentration in solution	Able to identify subpopulation of exosomes with specific protein markers	Resolution limit restricts the sensitivity, low amount of protein reduces the fluorescence signal for detection	(70)

## Application of Exosomal Proteins in Liquid Biopsy

The field of exosome research has put a huge effort into exploring exosomal miRNA as an attractive biomarker for cancer diagnosis because of the discovered correlation between the increased level of miRNA and the manifestation of cancer. However, it has been neglected that exosomal proteins can also serve as diagnostic biomarkers of cancer due to the following advantages: 1) protein constituents from tumor-derived exosomes reflect the proteomic profile of their parent tumor cells. Thus, circulating exosomes in the body fluids can provide comprehensive information about the distal primary tumor by the exosomal proteins (62). 2) Tumor-secreted proteins are greatly diluted by background substances in the circulating blood, making them very difficult to be detected. Enrichment of exosomes from blood can make it much easier to detect the proteins wrapped up in exosomes (13). 3) Many proteins secreted by tumor cells are unstable and vulnerable due to the presence of free protease in the body fluids. But these proteins can be well protected within tumor-derived exosomes, enabling in-depth analyses of relevant tumors (14). 4) Different miRNAs can lead to a similar consequence of gene expression sometimes (26), while the phenotypes of proteomic profiles can be more straightforward and representative of parent cells. Nevertheless, with increasing research on exosomal proteins for biomarker application, new challenges arise. For instance, the inconsistency among different cohorts of study is found even in the analysis of the same exosomal protein. Although there is still a long way to go, the development and application of exosomal protein as a powerful diagnostic tool for cancer therapy possess a bright future with tremendous potential. In this section, exosomal proteins that have been identified as tumor-associated markers by clinical research will be summarized and discussed. *In vitro* studies with cell culture or mouse models will not be covered because more attention has been paid to clinical studies, which present more relevant results to authentic physical situations. Exosomal proteins discussed here are summarized in **Tables 3**–**6**.

**Table 3:** Exosomal tetraspanin and surface markers.

Type of cancer	Protein marker	Number of patients/controls	Source of exosome/amount of Sample	Isolation technique	Diagnostic accuracy of proposed marker	Reference
**Lung cancer**	**CD91**	105/73	Serum/50 µl	MSIA (immunoaffinity capture)	AUC = 0.724, sensitivity = 60.0%, specificity = 89.0%.	(73)
					Control group includes 54 healthy individuals and 19 interstitial pneumonia patients.	
	**TSPAN8, CD151**	336/126	Plasma/10 µl	EV array (immunoaffinity capture)	AUC_TSPAN8_ = 0.60, AUC_CD151_ = 0.68. Patient group is composed of individuals with three types of lung cancer. Control group is composed of non-cancer patients having symptoms of cancer.	(74)
**Breast cancer**	**CD82**	80/80	Serum/500 µl	ExoQuick Kit (SBI)	Significantly increased in 30 representative samples.	(75)
					Control group is composed of patients with benign breast disease.	
**Colorectal cancer**	**CD9, CD147**	194/191	Serum/5 µl	ExoScreen (immunoaffinity capture)	AUC_CD9/CD147_ = 0.820.	(76)
					Double-positive exosomes were used as diagnostic markers.	
**Pancreatic cancer**	**TSPAN8**	131/79	Serum/1~1.5 ml	Sucrose gradient ultracentrifugation	Positive in 90% of patient samples.	(77)
					Control group include patients with chronic pancreatitis benign pancreatic tumor and non-pancreatic tumor and healthy volunteers.	
**Prostate cancer**	**CD9, CD81**	70/14	Plasma/over 3 ml	Ultracentrifugation	Significantly increased (CD9) in five representative patient samples. Significantly decreased (CD81) in four representative patient samples.	(78)
	**CD9**	6/10	Plasma/2,500 µg of protein	Ultracentrifugation/EV assay	Significantly increased in six representative samples.	(79)
					Control group is composed of patients with benign prostate hyperplasia.	
	**P-glycoprotein**	4/6	Serum/1 ml	Differential centrifugation	Increased in 4 docetaxel-resistant prostate cancer compared to 6 treatment-naïve patients.	(80)
**Melanoma**	**CD63**	90/58	Plasma/unspecified	Ultracentrifugation	Sensitivity = 96.5%, specificity = 43%. AUC unspecified.	(81)
**Oral squamous cell carcinoma**	**CD63**	10	Plasma/1 ml	Ultracentrifugation	Significantly decreased among 10 representative samples.	(82)
					Comparison was made between patients before and after surgery.	

EV, extracellular vesicle; AUC, area under the curve.

**Table 4:** Exosomal transport protein, heat shock protein, and adhesion protein.

Type of cancer	Protein marker	Number of patients/controls	Source of exosome/amount of sample	Isolation technique	Diagnostic accuracy of proposed marker	Reference
**Lung cancer**	**HSP70**	18/19	Plasma/10 ml blood	Ultracentrifugation	AUC = 0.8968 for distinguishing metastatic stage of cancer (including lung cancer and breast cancer). Comparison was made between patients with metastatic cancer and non-metastatic cancer.	(83)
	**Fibronectin**	21/41	Serum/unspecified	Sucrose gradient ultracentrifugation	AUC = 0.844.	(84)
**Gastric cancer**	**PSMA3/PSMA6**	24/13	Serum/2 ml	exoEasy Maxi Kit (QIAGEN)	Significantly increased in six patients with late-stage gastric cancer.	(85)
**Colorectal cancer**	**HSP90**	18/18	Serum/250 µl	Total Exosome Isolation Kit (Invitrogen)	Significantly decreased in 18 representative samples.	(86)
**Prostate cancer**	**αvβ3**	70/14	Plasma/over 3 ml	Ultracentrifugation	Significantly increased in representative samples.	(78)
	**ACTN4**	20/8	Serum/unspecified	Sucrose gradient ultracentrifugation	Significantly increased in castration-resistant prostate cancer compared to prostate cancer patients receiving primary androgen deprivation therapy.	(87)
**Pancreatic cancer**	**ZIP4**	24/78	Serum/500 µl	ExoQuick Kit (SBI)	AUC = 0.89 for distinguishing malignant cancer from benign pancreatic disease. AUC = 0.8931 for distinguishing malignant cancer group from normal group. [Number of controls include benign pancreatic disease (32) and normal subjects (46)]	(88)
**Endometrial cancer**	**ANXA2**	41/20	Plasma/500 µl	ExoGAG	AUC = 0.748.	(89)
**Gynecologic cancer**	**HSP22**	30	Serum/1 ml	Ultracentrifugation	Comparison was made among patients with ovarian cancer, patients with endometrial cancer and patients with endometriosis. Although no significant result was concluded.	(90)

If no specified AUC, sensitivity, or specificity is claimed in the reference, no precise numerical data for diagnostic accuracy are included in this table.

AUC, area under the curve.

**Table 5:** Tumor-associated exosomal protein.

Type of cancer	Protein marker	Number of patients/control	Source of exosome/amount of sample	Isolation technique	Diagnostic accuracy of proposed marker	Reference
**Glioblastoma**	**EGFR**	24/8	Plasma/unspecified	Differential centrifugation	AUC_EGFR_ = 0.78, sensitivity = 64.0%, specificity = 88.0%. AUC_EGFRVIII_ = 0.88, sensitivity = 68.0%, specificity = 100.0%.	(91)
**Lung cancer**	**EGFR**	9	Plasma/100 µl	Immunoaffinity capture	Positive in five representative samples.	(92)
**Hepatocellular carcinoma**	**LG3BP, PIGR**	29/32	Serum/1 ml	Ultracentrifugation	AUC_LG3BP_ = 0.904. Sensitivity = 96.6%, specificity = 71.8%. AUC_PIGR_ = 0.837. Sensitivity = 82.8%, specificity = 71.8%.	(93)
**Colorectal cancer**	**Glypican-1**	102/80	Plasma/10 ml blood	ExoCap™ kit (JSR)/immunoaffinity capture	Significantly decreased in cancer patients after surgery treatment compared to patients before surgery treatment. Significantly increased in patients compared to healthy control.	(94)
	**Glypican-1**	85	Plasma/20 ml blood	ExoCap™ kit (JSR)/immunoaffinity capture	Significantly decreased in cancer patients after surgery treatment compared to patients before surgery treatment. Significantly increased in late stage of cancer.	(95)
**Pancreatic cancer**	**c-Met**	30/40	Serum/250 µl	Total Exosome Isolation Kit (Invitrogen)	Sensitivity = 70%, specificity = 85%. Diagnostic odds ratio = 13:2. AUC_diagnostic_ not provided. AUC_prognostic_ = 0.779. Control group include non-malignant subjects, serous cystadenoma subjects, and chronic pancreatitis subjects.	(96)
	**Glypican-1**	62/20	Serum/250 µl	Sucrose gradient ultracentrifugation	AUC = 1.0. Sensitivity = 100.0%, specificity = 100.0%.	(97)
	**Glypican-1**	27/16	Plasma/1~1.5 ml	Ultracentrifugation	AUC = 0.59. Sensitivity = 74.0%, specificity = 44.0%. Control group is composed of patients with benign pancreatic disease.	(98)
	**Glypican-1**	24/26	Serum/2 ml	Sucrose gradient ultracentrifugation	AUC = 0.885.	(99)
**Pancreatic ductal adenocarcinoma**	**Glypican-1**	22/28	Serum/250 µl	Total Exosome Isolation Kit (Invitrogen)	AUC = 0.78 for GPC1^+^ exosomes in portal and peripheral blood. Sensitivity = 64.0%, specificity = 90.0%.	(100)
**Ovarian cancer**	**CD24, EpCAM, CA-125**	15/5	Plasma/20 µl	ExoSearch Chip/immunoaffinity capture	AUC_CD24_ = 0.9067. AUC_EpCAM_ = 1.000. AUC_CA-125_ = 1.000	(101)
**Melanoma**	**PD-L1**	44/11	Plasma/250 µl	TEI kit (Invitrogen)/ultracentrifugation	AUC = 0.9184. Sensitivity = 80.00%, specificity = 89.47%.	(102)
	**Caveolin-1**	90/58	Plasma/unspecified	Ultracentrifugation	Sensitivity = 69%, specificity = 96.3%.	(81)
**Head and neck squamous cell carcinomas**	**PD-L1**	40	Plasma/1 ml	Size exclusion chromatography/Immunoaffinity capture	Significantly increased in cancer patients with active disease and late stage (UICC stage III/IV) cancer.	(103)

If no specified AUC, sensitivity, or specificity is claimed in the reference, no precise numerical data for diagnostic accuracy are included in this table.

AUC, area under the curve.

**Table 6:** Tumor-associated exosomal protein in urine and ascites.

Type of Cancer	Protein Marker	Number of patients/controls	Source of exosome/amount of sample	Isolation technique	Diagnostic accuracy of proposed marker	Reference
**Prostate cancer**	**TMEM256**	16/15	Urine/50~150 ml	Ultracentrifugation	AUC = 0.87. Sensitivity = 94.0%, specificity = 100.0%.	(104)
**Bladder cancer**	**TACSTD2**	28/12	Urine/12.5 ml	Ultracentrifugation	AUC = 0.741. Control group is composed of 12 hernia patients. A higher AUC = 0.80 of TACSTD2 was obtained in a larger cohort of 221 samples with ELISA.	(105)
**Pancreatic cancer**	**CD133**	19	Ascites/unspecified	exoEasy Maxi Kit (QIAGEN)	The intensity of high-density glycosylation of CD133 significantly correlated with survival days of pancreatic patients. Non-malignant ascites from alcoholic and hepatitis C-related cirrhotic patients were considered as control.	(106)
**Endometriosis**	**ANXA2**	22/6	Peritoneal fluid/1 ml	Exo-spin Kit (Cell Guidance)	Specifically existed in endometriosis patients regardless of disease stage.	(18)
**Renal disease**	**Polycystin-1**	6	Urine/50 ml	Ultracentrifugation	Significantly increased in urinary exosomes. Comparison was made between urinary samples and kidney tissue samples.	(107)

If no specified AUC, sensitivity, or specificity is claimed in the reference, no precise numerical data for diagnostic accuracy are included in this table.

AUC, area under the curve.

### Tetraspanin and Surface Protein

Tetraspanins such as CD9, CD63, and CD81 are considered the major components of exosomal transmembrane proteins, which participate in the biogenesis and cargo sorting of exosomes (108). These three tetraspanins are also used most frequently as targets conjugated with the immunoaffinity capture method. Nevertheless, it has been noted that CD63 was less presented in all of the tested samples (109–111), which queries the widely accepted concept that CD63 is ubiquitously existed on the surface of exosomes and urges us to explore new exosomal signature proteins. Tumor-derived exosomes are known to play important roles in the process of tumor generation, progression, and EMT, as exosomal tetraspanins can interact with receptors like integrins on recipient cells for the enhanced uptake of tumor-derived exosomes. Hence, exosomes with increased expression of tetraspanins have been widely identified in the body fluids of cancer patients by different research groups. The correlation of tetraspanins with tumors also makes these exosomal surface proteins strong biomarkers for various cancers (112). An earlier proteomics study suggested that the expression of exosomal CD9 significantly varied among cancer tissues and relevant normal tissues (113). Recently, these proteins, including CD9, have been increasingly demonstrated to be closely relevant to cancer-derived exosomes by different groups, thus possessing tremendous potential as cancer biomarkers. An assay called ExoScreen was developed by the group of Yoshioka et al. (76) to screen exosomes with surface expressions of CD63 and CD9 from only 5 μl of serum from colorectal cancer patients without any sample preparation procedures such as dilution or purification. This assay combined two groups of antibodies conjugated with a donor photosensitizer bead and an acceptor photosensitizer bead. It was also found that the level of CD9 and another surface molecule CD147 was significantly increased in the serum of colorectal cancer patients (114). Notably, the higher level of CD147/CD9 in serum exosomes could also be detected at an early stage of cancer, with the area under the curve (AUC) of 0.820 among healthy donors versus patients. The regular reference tumor-associated antigens carcinoembryonic antigen (CEA) and carbohydrate antigen 19-9 (CA19-9) were within the normal value range (termed non-cancer) with the AUC of 0.669 and 0.622, respectively, indicating the great potential of exosomal proteins as cancer biomarkers with higher specificity and sensitivity than a regular signature. In another smaller cohort of studies about prostate cancer, Krishn et al. also confirmed that the CD9 level increased in plasma exosomes from patients compared to that of the healthy control group. Along with the upregulated CD9 levels on exosomes, the authors also found that αvβ3 integrin was also expressed in plasma exosomes of cancer patients, but the expression of another classic tetraspanin CD81 decreased somehow in cancer patients. However, a similar change was not found in the RNA level of CD9 and CD81 between healthy and disease conditions, suggesting that there should be an intriguingly complicated regulatory network involved in the process. Additionally, exosomal proteins should be more straightforward and convincing than exosomal RNA to reflect the cellular activity in a sense (78). Moreover, a higher expression of exosomal CD9 in plasma from prostate cancer patients had also been revealed in another study by differential centrifugation. However, these results came from a small-scale study with only six recruited patients and thus should be verified by identical experiments with a larger cohort. Nevertheless, they investigated the role of CD9 in prostate cancer and found that exosomal CD9 could promote cancer cell proliferation (79). An additional study on bladder cancer also reported an elevated level of CD9-positive exosomes in urinary samples (105). It looks like CD9 can promote tumor growth in different types of cancers, but an *in vivo* experiment has shown a contradictory result that the knockout of CD9 in hepatocellular carcinoma can facilitate cancer development instead. Additionally, it was also reported that there was no difference between the number of exosomes released by hepatocellular carcinoma cells before and after the overexpression of CD9 and CD81 (115). Although the results obtained from cell lines do not always display exactly the same in clinical scenarios, the complicated roles of CD9 discussed here should remind researchers that solid conclusions require more reliable comparative discoveries, especially regarding exosomes that are naturally heterogeneous.

Meanwhile, an in-house study of melanoma reported that there was a higher level of CD63^+^ exosomes in cancer plasma, which was correlative with the level of caveolin-1, a component of caveolae (81). Another pilot study with oral squamous cell carcinoma patients showed that the level of CD63^+^ exosomes decreased immediately after resection surgery, suggesting that the tumor was responsible for the high level of CD63^+^ exosomes in the circulation (82). In addition, to be potential candidates for cancer biomarkers, a recent study using immunohistochemistry (IHC) showed that tumor exosomal CD9 and CD63 might also act as potential prognostic monitors due to the increased expression in rectal tumor tissue after chemoradiation treatment (116). Although it is helpful to build our understanding of pathology by tissue biopsy, the result should be further verified with body fluid samples since liquid biopsy application of exosomes holds great promise in non-invasive personal precision medicine. So far, very few studies have investigated three tetraspanins (CD63, CD9, and CD81) together in clinical settings; therefore, it is still unclear whether the level of CD9, CD63, or CD81 correlates with different types of cancers.

Some other surface molecules expressed on exosomes were also reported as strong cancer biomarkers. In a large cohort study of lung cancer patients, an EV array was built that contained 49 exosomal and tumor proteins to collect the heterogeneous populations of exosomes from 472 isolated plasma samples. The expressions of two tetraspanins CD151 and TSPAN8 were found to be significantly elevated in exosomes from cancer patients, which could distinguish cancer from healthy control with AUC of 0.68 and 0.60, respectively, independently of disease stage and histological subtype. Following that, the combination of 8 proteins along with CD151 and TSPAN8 was determined to have the best separation outcome to distinguish cancer sample from healthy control with the largest AUC of 0.74, which suggested that a group of protein markers may have higher sensitivity than an individual marker in diagnosis (74). Another proteomic analysis presented similar data to show that the expression of exosomal TSPAN8 was associated with pancreatic cancer as well (77). A study on breast cancer demonstrated that the level of CD82 in serum-derived exosomes was significantly higher in a malignant group than in the healthy control group. The results also suggested that the level of CD82 was positively correlated with the malignancy of breast cancer and thus could sensitively serve as a breast cancer marker (75). There were also some other reports showing the increased level of exosomal CD91 and P-glycoprotein in the clinical case of lung cancer and prostate cancer, respectively (73, 80), indicating their potential for cancer analysis. Interestingly, the AUC of CD91 combined with CEA in distinguishing lung cancer reached 0.882 (73, 80), which substantiated CD91 as a very good candidate for potential lung cancer diagnosis.

There is a great chance that the surface molecules discussed above will be applied widely in future clinical research on cancer diagnosis and liquid biopsy, while most of these encouraging findings were involved with two correlated proteins, like CD9 and CD147 or CD63 and caveolin-1, because it may not be enough to get a whole picture of a tumor merely by a single protein marker. By combining various biomarkers in one panel, the signature of cancer can be identified and analyzed more reliably, accurately, and efficiently. Though promising, the results shown here came from different groups by using different purification processes without any normalization. Future studies with standardized methods are necessary to exclude the contingencies of experiments that might induce the methods of exosome enrichment.

### Transport Protein, Heat Shock Protein, and Adhesion Protein

TSG101, Alix, and heat shock proteins take part in the intracellular transport of exosomes, which therefore shows important implications in cancer (117–119). Elmira et al. reported in a recent study with over 400 samples that the level of exosomal TSG101 was significantly upregulated in colorectal tumor tissue (120). ANXA2, a member of the annexin family involved in the endocytosis and exocytosis processes, was identified to have an increasing level in plasma exosomes of endometrial cancer (EC) patients. The AUC of ANXA2 as a biomarker for EC is 0.74, which therefore can serve as a relatively good marker suggested by the authors (89). HSP70 is actively released as exosome surface protein under the stimulation of high levels of IL-10 and IFN-γ in the serum of cancer patients, suggesting the role of exosomal HSP70 in tumor immunity and the potential of HSP70 as a cancer biomarker (121). Moreover, it was found that the number of HSP70^+^ exosomes in the plasma samples from metastatic lung and breast cancer patients was significantly higher than that of healthy volunteers, in which the level of HSP70^+^ exosomes was barely detectable (83). The same group further compared the performance of exosomal HSP70 vs. CTCs in their pilot attempt under clinical conditions and found that exosomal HSP70 could act as a discriminative marker between patients with non-metastatic lung cancer and metastatic lung cancer, possessing better AUC of 0.8968 than that of CTC detection (AUC = 0.7857). Under stress, the induced form of HSP70 is expressed exclusively by cancer cells, which is called HSP72. One of the conclusive reviews of HSP70 discussed that tumor-derived HSP72-containing exosomes promoted the immunosuppressive function of tumor-associated suppressor cells, indicating the diagnostic value of HSP72 as the target of cancer (121, 122). Other than HSP70, an integrin-mediated pathway-related heat shock protein HSP90 was also reported to be strongly downregulated in the serum of colorectal cancer patients compared to the healthy control group (86). Moreover, one of small heat shock proteins (sHSP), named HSP22, was found at a high level in exosomes from gynecologic cancer patients, which was further suggested to be correlated with host cytotoxic immune response (90).

Subunits PSMA3 and PSMA6, belonging to the 26S proteasome complex that is responsible for the functional modification and the degradation of cellular proteins, were shown to be enriched in serum-derived exosomes of gastric cancer patients (85). In addition, exosomal zinc transporter ZIP4 and integrin αvβ3 were also reported to be significantly related to tumor growth of pancreatic and prostate cancers, respectively (78, 88). In the latter study, to determine the origin of exosomes, C4-2B-β3-GFP cells were injected into NOD mice to develop prostate cancer, and the authors successfully isolated plasma exosomes with GFP-tagged αvβ3 integrin in the circulation, indicating the direct relevance between tissue-derived exosomes and circulating exosomes (78). Moreover, exosomal fibronectin was also confirmed by proteomic analysis on non-small cell lung cancer-derived exosomes to display great diagnostic potential in the clinical cohort (84). The actin cross-linking protein actinin-4, which could facilitate cancer metastasis, was also identified as a signature protein of metastatic prostate cancer with a high expression level (87).

### Tumor-Specific Protein

Tumor-associated proteins such as CA19-9, CEA, and prostate-specific antigen (PSA) have been used as indicators in cancer diagnosis for a long time. Among those significant biomarkers, PD-L1 has become the focus of immunotherapy development. PD-L1, existing on the surface of various cell types including epithelial cells and tumor cells, can bind with PD-1 on the surface of T cells to act as the key immune checkpoint mediator in healthy conditions; however, it can also suppress the immune response of cytotoxic T cells in cancer. Overexpression of PD-L1 has been reported to be indicative of the status of tumors by using methods like IHC (123). Compared to tissue biopsy, liquid biopsy offers a safer way of sampling, which can be conducted at any time to acquire accurate information about the ongoing illness in patients. Due to that, research starts to focus on the investigation of the value of exosomal PD-L1 in liquid biopsy. A mechanism study has demonstrated that metastatic melanoma cells managed to escape from immune supervision by producing exosomes that carry PD-L1 to facilitate tumor growth as well as suppress CD8^+^ T cells. Additionally, the authors also found that compared to that in healthy donors, the level of PD-L1 in plasma exosomes was significantly higher in melanoma patients (102). Similarly, in patients with head and neck cancer, the expression of PD-L1 on the membrane of plasma exosomes was discovered to be associated with the progression of the disease, whereas soluble PD-L1 was not correlated with any of the results found with exosomal surface PD-L1 (103). This odd observation may suggest that the exosomal surface proteins exhibit the same expression pattern as parental cells. Nevertheless, a study on pancreatic cancer revealed controversial results showing that the level of PD-L1 on circulating exosomes was not correlated with cancer. On the contrary, another molecule c-Met displayed a strong correlation with cancerous parameters (96). Thus, based on these favorable findings, more future work should be done to provide convincing proof that exosomal PD-L1 can be developed as an excellent cancer biomarker.

Glypican-1 (GPC-1), a surface-bound proteoglycan that regulates signaling pathways mediated by TGF-β, Wnt, and other growth factors, is overexpressed in a variety of cancer tissues to promote cancer progression, especially pancreatic cancer (124, 125). Melo et al. purified serum exosomes from patients with pancreatic ductal adenocarcinoma (PDAC) and found that all exosome samples of PDAC patients (n = 190) were marked with high expression of GPC-1, suggesting a strong correlation between cancer and serum-derived exosomes. More than that, the authors analyzed the primary tumor tissue of PDAC patients and found the mutant transcript of Kras, an oncogenic gene that is frequently seen in PDAC. They confirmed the presence of similar Kras mutation in GPC-1^+^ serum exosomes of PDAC patients. These results indicated that a part of circulating exosomes was derived from relevant tumors, which underscored the importance of exosomal GPC-1 as a pancreatic cancer biomarker (97). A similar study with PDAC further emphasized the potential of exosomal GPC-1 by showing that the level of GPC-1 was elevated in plasma exosomes of PDAC patients, which was decreased accordingly after the resection surgery of PDAC patients (98). However, in many studies with PDAC, the investigation of exosomal GPC-1 was usually combined with other surface markers, which were claimed to possess higher sensitivity and specificity for cancer detection. Buscail et al. reported that the combination with CA19-9 and CD63^+^ GPC-1^+^ circulating exosomes could define PDAC much better with an accuracy reaching 84% (100). Xiao et al. also addressed that a sensitive and reproducible detection panel consisting of exosomal GPC-1 and CD82 and serum CA19-9 could efficiently distinguish PDAC patients from healthy people (99). Furthermore, a multiparametric exosome profiling was also developed and conducted by screening exosomes from patient plasma with a combination of ten surface markers including four biomarkers from a major group of cancers, three putative PDAC markers including GPC-1, and three pan-exosome markers (126). Exosomal GPC-1 has also been demonstrated to be increased in colorectal cancer (94). The percentage of GPC-1^+^ exosomes among total exosome numbers was significantly higher in patients with more serious disease conditions (95). This percentage was also significantly increased in relapsed patients, indicating GPC-1 as a biomarker for diagnosis and poor prognosis (95). Collectively, these research data obtained with clinical samples greatly support the value of exosomal GPC-1 as a potential diagnostic marker for cancer screening.

CD24 is a type of cell adhesion molecule participating in cell recognition, activation, signal transduction, proliferation, etc., which has been found to be abnormally expressed in various cancers. Notably, Zhao et al. developed a multiplexed detection chip combined with protein markers CA-125, EpCAM, and CD24, which indicated a three-fold increase of exosomal CD24 in the plasma of ovarian cancer patients. The AUC values of CA-125, EpCAM, and CD24 were 1.0, 1.0, and 0.91, respectively, all indicating the great value of diagnosis (101). But Soltész et al. found that in the same type of cancer, the expression of CD24 RNA was not detectable in all plasma or exosome samples of the patients, though there is a significant alteration in the tissue of the same ovarian cancer patients (127). Thus the bias existing between exosomal protein and RNA remains to be solved, and it also signifies the importance of exosomal protein in liquid biopsy. Epidermal growth factor receptor (EGFR), another protein specifically overexpressed in tumors, is closely associated with cancer progression. Yamashita et al. showed that the expression of EGFR on plasma exosomes was significantly higher in lung cancer patients compared to healthy controls, whereas the level of soluble EGFR in plasma showed no significant difference between healthy and disease conditions (92). In another study, a microfluidic chip combined with a nuclear magnetic resonance detection system was developed by Shao et al. to analyze the expressions of EGFR and its variant EGFR vIII on plasma exosomes of glioblastoma patients (91). They identified that the levels of EGFR and EGFRvIII in exosomes were significantly higher in patients compared to healthy individuals.

Survivin-2B, a pro-apoptotic protein mainly found in primary tumors rather than high-grade tumors, could be further developed as a diagnostic marker for breast cancer (128). Caveolin-1, referred to in the above section as exosomal CD63, was reported to be increased in plasma-derived exosomes of melanoma patients, with even higher sensitivity than CD63 as a putative melanoma biomarker (81). Galectin-3-binding protein (LG3BP), a protein promoting tumor growth, was found in the serum-derived exosomes of hepatocellular carcinoma (HCC; liver cancer) patients with remarkably increased expression compared to that of the healthy control group. It was worth noting that although exosomal miRNAs in liver cancer were widely studied, there was a paucity of studies with exosomal protein biomarkers, which urged researchers to pay more attention to potential protein biomarkers for liver cancer. The diagnostic AUC of LG3BP was 0.904, which was much higher than that of a non-specific tumor marker alpha-fetoprotein (AUC = 0.802). However, another tumor marker PIGR was surprisingly found to be elevated in serum-derived exosomes with the diagnostic AUC of 0.837, higher than that of alpha-fetoprotein (93). Thus, LG3BP and PIGR may work as novel biomarkers for liver cancer together. Common cancer like lung cancer and breast cancer are both discussed in this review regarding different cancer-associated exosomal protein markers. However, rather than exosomal protein, there are much more research data in the clinical field of HCC that showed nucleic acids in exosomes including miRNA and lncRNA can act as novel biomarkers (129).

Exosomal proteins discussed in the above section almost came from the study with either plasma or serum. Other body fluids like urine or ascites, which are less viscous than plasma or serum, are much easier to deal with. Exosomes from these sources also have been revealed as potential tools for diagnosis (31). For example, a proteome profile study of urine-derived exosomes identified proteins from renal tubule epithelial cells. Polycystin-1, specifically identified in urine-derived exosomes, was associated with multiple renal diseases (107). The same group further developed an efficient protocol for urinary exosome isolation and storage (130). Different from plasma or serum-derived exosomes, urine-derived exosomes are sometimes more relevant to the urinary system, which suggests that they might possess greater potential as early liquid biopsy biomarkers for renal-related diseases. In urological cancers like prostate cancer or bladder cancer, urine-derived exosomes still display great value as diagnosis signature. One of the comprehensive proteomic studies of prostate cancer described in a review (104) suggested that TMEM256 displayed excellent accuracy for cancer diagnosis with a high AUC of 0.94 in clinical urinary samples. Nevertheless, in the discussion of another review, though another group proposed TMEM256 as a potential biomarker for prostate cancer, two other groups could not detect the presence of TMEM256 in two sets of clinical urinary samples. Moreover, the protein profiles obtained by these two groups in the urinary sample of prostate cancer were also distinct, possibly due to the different isolation and quantification methods or a different control group (131).

Moreover, there was also a study by the group of Chen et al. about a potential biomarker TACSTD2 for bladder cancer, which was significantly increased in urinary exosomes of patients compared to individuals with hernia (105). The great potential of TACSTD2 as a diagnostic biomarker for bladder cancer was further supported by the validation of another cohort of study (131) and by the fact that it was exclusively made by cancer cells (132). Ascite-derived exosomes are also found as a strong diagnostic tool for their expression of highly glycosylated CD133 in pancreatic cancer (106). Meanwhile, the group of Nazri et al. discovered the connection between peritoneal fluid-derived exosomes and endometriosis by systemic liquid chromatography with tandem mass spectrometry and identified five associated exosomal proteins including AXNA2 to be potential diagnostic biomarkers for endometriosis (18). The discussion about tumor-associated exosomal proteins as potential cancer biomarkers should also remind researchers of the fact that the clinical data are still limited. For example, when investigating prostate cancer, exosomal proteins including CD9, CD81, P-glycoprotein, and ACTN4 together can act as a detection panel with high specificity. CD9, CD63, or glypican-1 can be taken into consideration as generic tumor-associated markers of exosomes produced by a major group of cancers. In the future, more comprehensive research should be conducted in representative clinical cohorts to further substantiate the existing data.

## Conclusion and Perspective

With the increasing knowledge of exosomal functions in cell–cell communication, the application of exosomal proteins as a potential tool for cancer diagnosis and liquid biopsy has become more and more attractive. Under normal circumstances, tissue biopsy or pulmonary lavage is carried out to examine the state of lung cancer, which undoubtedly causes damage to the body. Notably, in a proteomic study of non-small cell lung cancer, Li et al. demonstrated that AQP-2 (kidney tissue-related)-positive urinary exosomes expressed a high level of LRG1 protein. Similarly, LRG1 was also found highly expressed in the lung tissue of cancer patients (133). The study has presented us with a clue to the pulmonary origin of exosomes dispersed in urine and a special perspective on diagnostic utilization of urinary exosomes for lung cancer. Moreover, the discovered correlations between urine-derived exosomes and distant cancerous lung tissue encourage researchers to look deeper into the origin, transport, circulation, and uptake of exosomes.

**Figure 3:**
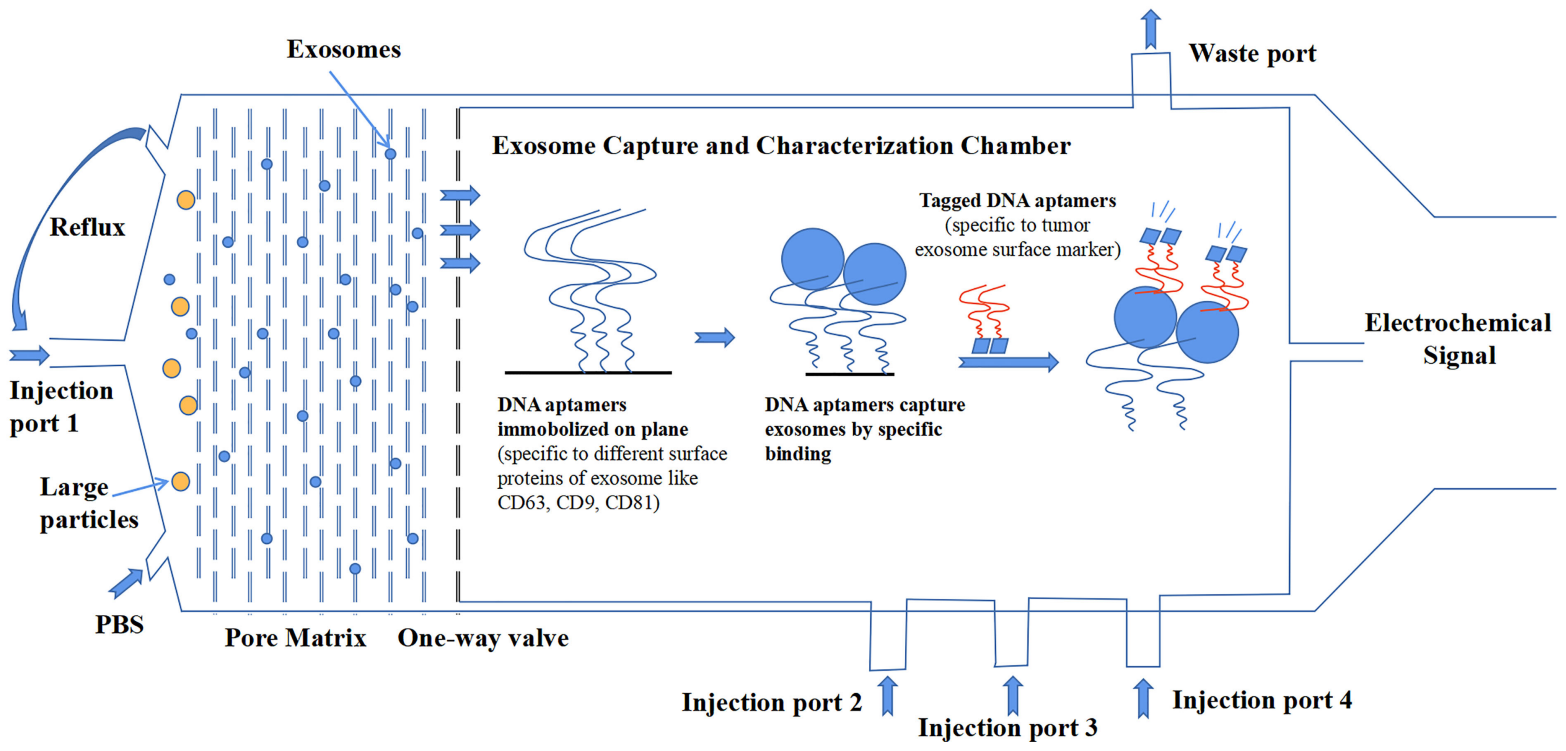
A design of microfluidics system for clinical exosome isolation and characterization with high efficiency and high sensitivity. The first module of the system is a pore matrix (pore size ~200 nm). When microliters of plasma/serum is injected through the injection port 1, pressure will be added to the module, which makes particles inside the sample move toward the pore matrix. Nanoparticles including exosomes will move through pore matrix, while large particles will be left in the chamber. Then, phosphate-buffered saline (PBS) solution is injected into the chamber to create reflux to wash the pore matrix to ensure all exosomes move across the matrix and reach the one-way valve. In the second module, exosomes were captured in a characterization chamber. Enough quantities of DNA aptamers that specifically recognize exosome surface proteins like CD63, CD81, and CD9 are immobilized on the plane. Exosomes were captured while flowing across the plane, and buffer was injected through port 2. Impurities were discharged through the waste port. After that, tagged DNA aptamers that specifically recognize tumor-specific antigens were injected through port 3, and tumor-derived exosomes were bound by those aptamers. Finally, the chemiluminescence reagent was injected through port 4 and reacted with the tagged aptamers. The chemical signal was captured and exported eventually.

Compared to the detection of tumors by classic antigens, the combination of exosomal protein markers offers better diagnostic results (76). Although increasing research data have suggested the importance of exosomal proteins, there are also some inconsistent and paradoxical results that we have discussed in this review among different studies, which urge a more precise investigation of exosomes. The most important reason for this result is that no standardized methodology of isolation and characterization has been established in the whole field of exosome research. Additionally, exosomes are heterogeneous, which makes the creation of standardized methods very necessary to promote the development of the clinical application of exosomal proteins. In a recent comparative study on the tetraspanin profile of exosomes, the heterogeneity of exosomes was underlined to show a strong impact on the diagnostic sensitivity of exosomal surface markers. Good consistency exists in the tetraspanin profile of exosomes from the same source regardless of isolation methods. But the results confirmed that tetraspanin profiles of exosomes from a different source are distinct (134). Additionally, other exosomal cargos can also differ among different sources. From the long-term point of view, the development of a microfluidics system is more likely to be rapidly upgraded because it represents the cutting-edge technology that can satisfy the needs of exosome investigation. Microfluidics can integrate separate isolation procedures and characterization procedures into a single platform that skips the conservation of exosomes. Even more encouraging, it can analyze multiple samples that have a small volume to detect exosomes with high throughput and high precision (135). **Figure 3** shows a microfluidic system conceived by our group that delivers a general workflow or construction that can isolate and characterize tumor-derived exosomes in clinical samples.

In a recent study, Zhang et al. presented a microfluidic system in which exosomes are captured by an anti-CD63 aptamer and diagnosed by an anti-EpCAM aptamer (136). To detect those captured exosomes, a hybridization chain reaction (HCR) is applied. In this way, the signal of EpCAM aptamer on the surface of CD63^+^ exosomes is amplified in a linear form that can be detected with high sensitivity. The detection limit is confirmed as 0.5 exosomes/µl, and Zhang et al. also compared the performance of the system to that of NTA, which showed that the system could isolate cancer-derived exosomes and spare non-cancerous exosomes at the same time. Notably, a similar microfluidic aptasensor (aptamer-combined sensor) platform was constructed by Wang et al. to detect tumor-derived exosomes (137). The principle of HCR was also used, but in a different way to accurately amplify the signal of EpCAM^+^ exosomes by the formation of a multidirectional reaction. Surprisingly, the detection limit reached 285 exosomes/µl, significantly higher than that of other systems. The huge difference that existed between the two systems suggested that the mechanism applied for the amplification and detection of signals in an aptasensor system had a great impact on the sensitivity. However, a remaining issue of these two studies is that the exosomes they used for testing are enriched either by ultracentrifugation of supernatant of cell culture or by isolation of human serum with commercial isolation kit. Therefore, the detection sensitivity and limit of these two systems for exosomal EpCAM diagnosis of more complicated clinical samples without exosome enrichment remain unknown. Moreover, in these two studies, the use of a single biomarker CD63 for exosome capture resulted in the omission of exosomes deprived of surface CD63. Similarly, use of single biomarker EpCAM resulted in the omission of tumorous exosomes lack of EpCAM.

While these issues are taken into consideration in our proposed model (**Figure 3**), the combination of multiple exosomal surface markers in this model can promote the probability to capture almost all exosomes in samples; however, the actual performance of the device still relies on the specificity of current exosomal markers and the identification of novel exosomal markers. Moreover, the sensitivity for detecting or capturing tumor-derived exosomes by the model also relies on the binding affinity of tumor-associated aptamers to exosomes, as well as the technique applied. The exclusion of non-cancerous exosomes would be favored by the technical improvement of detection strategy and the discovery of some novel tumor markers with high specificity, which would result in the enrichment of cancerous exosomes. For clinical use of exosomes, the methods should be handy, highly efficient, and supersensitive. The methods should also be compatible with different clinical samples. When dealing with blood, coagulation will cause the release of platelet-derived exosomes in the serum; thus it is better to choose plasma for exosome isolation (138). However, coagulation sometimes happens with cancer progression; thus, it is extremely necessary to distinguish tumor-derived exosomes from immune cell-derived exosomes in the blood sample, which further emphasizes the need for the discovery of tumor-specific exosomal markers. Additionally, the development of cancer-stage-specific exosomal markers is also very attractive to the current precision medicine theory.

It is worth mentioning that the standardization of exosome isolation methods will favor future clinical utilization of exosomes for immunotherapy at the same time. Safety is the most important issue to be concerned about when talking about therapy, which holds back many cell-therapy strategies. On the contrary, the high purity of exosome products will be a strong candidate for therapeutic treatment. For example, mesenchymal stem cell (MSC)-derived exosomes have already been proved to mediate immune response in multiple murine models with acute graft-versus-host disease (aGVHD) due to their anti-inflammatory or immunomodulatory effects. MSC-derived exosomes can promote the expansion of regulatory T cells, which suppress the inflammatory response and can also induce the production of anti-inflammatory cytokines like IL-10 while inhibiting the production of inflammatory cytokines like TNF-α (139, 140). In a typical clinical treatment against aGVHD, the response of patients to MSC-derived exosomes administration was found to be positive, and the clinical GVHD symptom was significantly improved, suggesting the great potential of MSC-derived exosomes for clinical therapies (141). Beyond that, the function of MSC-derived exosomes has also been recently introduced into the treatment of COVID-19 for the first time. Patients infected with COVID-19 received 5 days of MSC-derived exosome infusion. At the endpoint, data showed that the percentage of CD3^+^, CD4^+^, and CD8^+^ T cells increased in patients, implying the exciting outcome of immunomodulatory effects of MSC-derived exosomes (142).

In summary, it is believed that with the advancement of isolation technique and characterization strategy, the clinical application of exosomal proteins would finally act as a powerful diagnostic target in liquid biopsy as well as effective immunotherapy.

## Author Contributions

CH and WJ drafted the manuscript. ML, SF, and YL helped to revise the manuscript. JP and QW were responsible for leading this work and revising the manuscript. All authors listed have made a substantial, direct, and intellectual contribution to the work and approved it for publication.

## Funding

This study was supported by the Open Research Fund of Songshan Lake Materials Laboratory (2021SLABFN10),the Natural Science Foundation of Guangdong Province(2022A1515011223), Project of Educational Commission of Guangdong Province of China (2021KTSCX038), and Funds for PhD researchers of Guangdong Medical University in 2021(4SG21236G and 4SG22067G).

## Conflict of Interest

The authors declare that the research was conducted in the absence of any commercial or financial relationships that could be construed as a potential conflict of interest.

## Publisher’s Note

All claims expressed in this article are solely those of the authors and do not necessarily represent those of their affiliated organizations, or those of the publisher, the editors and the reviewers. Any product that may be evaluated in this article, or claim that may be made by its manufacturer, is not guaranteed or endorsed by the publisher.

## References

[1] Oshi M Murthy V Takahashi H Huyser M Okano M Tokumaru Y. Urine as a Source of Liquid Biopsy for Cancer. Cancers (Basel) (2021) 13(11):2652. doi: 10.3390/cancers13112652 34071230 PMC8199052

[2] Watanabe K Nakamura Y Low SK. Clinical Implementation and Current Advancement of Blood Liquid Biopsy in Cancer. J Hum Genet (2021) 66(9):909–26. doi: 10.1038/s10038-021-00939-5 34088974

[3] Wu L Wang Y Zhu L Liu Y Wang T Liu D. Aptamer-Based Liquid Biopsy. ACS Appl Bio Materials (2020) 3(5):2743–64. doi: 10.1021/acsabm.9b01194 35025406

[4] Gormally E Hainaut P Caboux E Airoldi L Autrup H Malaveille C. Amount of DNA in Plasma and Cancer Risk: A Prospective Study. Int J Cancer (2004) 111(5):746–9. doi: 10.1002/ijc.20327 15252845

[5] Sharma S Zhuang R Long M Pavlovic M Kang Y Ilyas A. Circulating Tumor Cell Isolation, Culture, and Downstream Molecular Analysis. Biotechnol Adv (2018) 36(4):1063–78. doi: 10.1016/j.biotechadv.2018.03.007 PMC5971144 29559380

[6] Wu CP Wu P Zhao HF Liu WL Li WP. Clinical Applications of and Challenges in Single-Cell Analysis of Circulating Tumor Cells. DNA Cell Biol (2018) 37(2):78–89. doi: 10.1089/dna.2017.3981 29265876

[7] Diehl F Li M Dressman D He Y Shen D Szabo S. Detection and Quantification of Mutations in the Plasma of Patients With Colorectal Tumors. Proc Natl Acad Sci USA (2005) 102(45):16368–73. doi: 10.1073/pnas.0507904102 PMC1283450 16258065

[8] Schwarzenbach H Hoon DS Pantel K. Cell-Free Nucleic Acids as Biomarkers in Cancer Patients. Nat Rev Cancer (2011) 11(6):426–37. doi: 10.1038/nrc3066 21562580

[9] Ko J Carpenter E Issadore D. Detection and Isolation of Circulating Exosomes and Microvesicles for Cancer Monitoring and Diagnostics Using Micro-/Nano-Based Devices. Analyst (2016) 141(2):450–60. doi: 10.1039/c5an01610j PMC4881422 26378496

[10] Valencia K Montuenga LM. Exosomes in Liquid Biopsy: The Nanometric World in the Pursuit of Precision Oncology. Cancers (Basel) (2021) 13(9):2147. doi: 10.3390/cancers13092147 33946893 PMC8124368

[11] Vaidyanathan R Soon RH Zhang P Jiang K Lim CT. Cancer Diagnosis: From Tumor to Liquid Biopsy and Beyond. Lab Chip (2018) 19(1):11–34. doi: 10.1039/c8lc00684a 30480287

[12] Lombardi CP Bossola M Princi P Boscherini M La Torre G Raffaelli M. Circulating Thyroglobulin mRNA Does Not Predict Early and Midterm Recurrences in Patients Undergoing Thyroidectomy for Cancer. Am J Surg (2008) 196(3):326–32. doi: 10.1016/j.amjsurg.2007.09.047 18614150

[13] Zhou B Xu K Zheng X Chen T Wang J Song Y. Application of Exosomes as Liquid Biopsy in Clinical Diagnosis. Signal Transduct Target Ther (2020) 5(1):144. doi: 10.1038/s41392-020-00258-9 32747657 PMC7400738

[14] Li W Li C Zhou T Liu X Liu X Li X. Role of Exosomal Proteins in Cancer Diagnosis. Mol Cancer (2017) 16(1):145. doi: 10.1186/s12943-017-0706-8 28851367 PMC5576100

[15] Fitts CA Ji N Li Y Tan C. Exploiting Exosomes in Cancer Liquid Biopsies and Drug Delivery. Adv Healthc Mater (2019) 8(6):e1801268. doi: 10.1002/adhm.201801268 30663276

[16] Liu Y Shi K Chen Y Wu X Chen Z Cao K. Exosomes and Their Role in Cancer Progression. Front Oncol (2021) 11:639159. doi: 10.3389/fonc.2021.639159 33828985 PMC8020998

[17] Thery C Witwer KW Aikawa E Alcaraz MJ Anderson JD Andriantsitohaina R. Minimal Information for Studies of Extracellular Vesicles 2018 (MISEV2018): A Position Statement of the International Society for Extracellular Vesicles and Update of the MISEV2014 Guidelines. J Extracell Vesicles (2018) 7(1):1535750. doi: 10.1080/20013078.2018.1535750 30637094 PMC6322352

[18] Nazri HM Imran M Fischer R Heilig R Manek S Dragovic RA. Characterization of Exosomes in Peritoneal Fluid of Endometriosis Patients. Fertil Steril (2020) 113(2):364–73 e2. doi: 10.1016/j.fertnstert.2019.09.032 32106990 PMC7057257

[19] Kalluri R LeBleu VS. The Biology, Function, and Biomedical Applications of Exosomes. Science (2020) 367(6478):eaau6977. doi: 10.1126/science.aau6977 32029601 PMC7717626

[20] van Niel G D'Angelo G Raposo G. Shedding Light on the Cell Biology of Extracellular Vesicles. Nat Rev Mol Cell Biol (2018) 19(4):213–28. doi: 10.1038/nrm.2017.125 29339798

[21] Gurunathan S Kang MH Qasim M Khan K Kim JH. Biogenesis, Membrane Trafficking, Functions, and Next Generation Nanotherapeutics Medicine of Extracellular Vesicles. Int J Nanomedicine (2021) 16:3357–83. doi: 10.2147/IJN.S310357 PMC8140893 34040369

[22] Thery C Amigorena S Raposo G Clayton A. Isolation and Characterization of Exosomes From Cell Culture Supernatants and Biological Fluids. Curr Protoc Cell Biol (2006) 93:3.22.1-29. doi: 10.1002/0471143030.cb0322s30 18228490

[23] Keerthikumar S Chisanga D Ariyaratne D Al Saffar H Anand S Zhao K. ExoCarta: A Web-Based Compendium of Exosomal Cargo. J Mol Biol (2016) 428(4):688–92. doi: 10.1016/j.jmb.2015.09.019 PMC4783248 26434508

[24] Reddy VS Madala SK Trinath J Reddy GB. Extracellular Small Heat Shock Proteins: Exosomal Biogenesis and Function. Cell Stress Chaperones (2018) 23(3):441–54. doi: 10.1007/s12192-017-0856-z PMC5904088 29086335

[25] Goldie BJ Dun MD Lin M Smith ND Verrills NM Dayas CV. Activity-Associated miRNA Are Packaged in Map1b-Enriched Exosomes Released From Depolarized Neurons. Nucleic Acids Res (2014) 42(14):9195–208. doi: 10.1093/nar/gku594 PMC4132720 25053844

[26] Valadi H Ekstrom K Bossios A Sjostrand M Lee JJ Lotvall JO. Exosome-Mediated Transfer of mRNAs and microRNAs Is a Novel Mechanism of Genetic Exchange Between Cells. Nat Cell Biol (2007) 9(6):654–9. doi: 10.1038/ncb1596 17486113

[27] Salehi M Sharifi M. Exosomal miRNAs as Novel Cancer Biomarkers: Challenges and Opportunities. J Cell Physiol (2018) 233(9):6370–80. doi: 10.1002/jcp.26481 PMC13482446 29323722

[28] Chatterjee N Rana S Espinosa-Diez C Anand S. MicroRNAs in Cancer: Challenges and Opportunities in Early Detection, Disease Monitoring, and Therapeutic Agents. Curr Pathobiol Rep (2017) 5(1):35–42. doi: 10.1007/s40139-017-0123-0 28966883 PMC5613763

[29] Chargaff E West R. The Biological Significance of the Thromboplastic Protein of Blood. J Biol Chem (1946) 166(1):189–97. doi: 10.1016/S0021-9258(17)34997-9 20273687

[30] Johnstone RM Adam M Hammond JR Orr L Turbide C. Vesicle Formation During Reticulocyte Maturation. Association of Plasma Membrane Activities With Released Vesicles (Exosomes). J Biol Chem (1987) 262(19):9412–20. doi: 10.1016/S0021-9258(18)48095-7 3597417

[31] Keller S Ridinger J Rupp AK Janssen JW Altevogt P. Body Fluid Derived Exosomes as a Novel Template for Clinical Diagnostics. J Transl Med (2011) 9:86. doi: 10.1186/1479-5876-9-86 21651777 PMC3118335

[32] Wei Y Lai X Yu S Chen S Ma Y Zhang Y. Exosomal miR-221/222 Enhances Tamoxifen Resistance in Recipient ER-Positive Breast Cancer Cells. Breast Cancer Res Treat (2014) 147(2):423–31. doi: 10.1007/s10549-014-3037-0 25007959

[33] Tian T Zhu YL Zhou YY Liang GF Wang YY Hu FH. Exosome Uptake Through Clathrin-Mediated Endocytosis and Macropinocytosis and Mediating miR-21 Delivery. J Biol Chem (2014) 289(32):22258–67. doi: 10.1074/jbc.M114.588046 PMC4139237 24951588

[34] Parolini I Federici C Raggi C Lugini L Palleschi S De Milito A. Microenvironmental pH Is a Key Factor for Exosome Traffic in Tumor Cells. J Biol Chem (2009) 284(49):34211–22. doi: 10.1074/jbc.M109.041152 PMC2797191 19801663

[35] Kitai Y Kawasaki T Sueyoshi T Kobiyama K Ishii KJ Zou J. DNA-Containing Exosomes Derived From Cancer Cells Treated With Topotecan Activate a STING-Dependent Pathway and Reinforce Antitumor Immunity. J Immunol (2017) 198(4):1649–59. doi: 10.4049/jimmunol.1601694 28069806

[36] Sheller-Miller S Trivedi J Yellon SM Menon R. Exosomes Cause Preterm Birth in Mice: Evidence for Paracrine Signaling in Pregnancy. Sci Rep (2019) 9(1):608. doi: 10.1038/s41598-018-37002-x 30679631 PMC6345869

[37] Vincent-Schneider H Stumptner-Cuvelette P Lankar D Pain S Raposo G Benaroch P. Exosomes Bearing HLA-DR1 Molecules Need Dendritic Cells to Efficiently Stimulate Specific T Cells. Int Immunol (2002) 14(7):713–22. doi: 10.1093/intimm/dxf048 12096030

[38] Qu JL Qu XJ Zhao MF Teng YE Zhang Y Hou KZ. Gastric Cancer Exosomes Promote Tumour Cell Proliferation Through PI3K/Akt and MAPK/ERK Activation. Dig Liver Dis (2009) 41(12):875–80. doi: 10.1016/j.dld.2009.04.006 19473897

[39] Yang L Wu XH Wang D Luo CL Chen LX. Bladder Cancer Cell-Derived Exosomes Inhibit Tumor Cell Apoptosis and Induce Cell Proliferation *In Vitro* . Mol Med Rep (2013) 8(4):1272–8. doi: 10.3892/mmr.2013.1634 23969721

[40] Aga M Bentz GL Raffa S Torrisi MR Kondo S Wakisaka N. Exosomal HIF1alpha Supports Invasive Potential of Nasopharyngeal Carcinoma-Associated LMP1-Positive Exosomes. Oncogene (2014) 33(37):4613–22. doi: 10.1038/onc.2014.66 PMC4162459 24662828

[41] Yoshizaki T Kondo S Wakisaka N Murono S Endo K Sugimoto H. Pathogenic Role of Epstein-Barr Virus Latent Membrane Protein-1 in the Development of Nasopharyngeal Carcinoma. Cancer Lett (2013) 337(1):1–7. doi: 10.1016/j.canlet.2013.05.018 23689138

[42] Fiaschi T Giannoni E Taddei ML Cirri P Marini A Pintus G. Carbonic Anhydrase IX From Cancer-Associated Fibroblasts Drives Epithelial-Mesenchymal Transition in Prostate Carcinoma Cells. Cell Cycle (2013) 12(11):1791–801. doi: 10.4161/cc.24902 PMC3713137 23656776

[43] Yue S Mu W Erb U Zoller M. The Tetraspanins CD151 and Tspan8 Are Essential Exosome Components for the Crosstalk Between Cancer Initiating Cells and Their Surrounding. Oncotarget (2015) 6(4):2366–84. doi: 10.18632/oncotarget.2958 PMC4385857 25544774

[44] Berchem G Noman MZ Bosseler M Paggetti J Baconnais S Le Cam E. Hypoxic Tumor-Derived Microvesicles Negatively Regulate NK Cell Function by a Mechanism Involving TGF-Beta and Mir23a Transfer. Oncoimmunology (2016) 5(4):e1062968. doi: 10.1080/2162402X.2015.1062968 27141372 PMC4839360

[45] Taylor DD Gercel-Taylor C. Exosomes/microvesicles: Mediators of Cancer-Associated Immunosuppressive Microenvironments. Semin Immunopathol (2011) 33(5):441–54. doi: 10.1007/s00281-010-0234-8 21688197

[46] Ying X Wu Q Wu X Zhu Q Wang X Jiang L. Epithelial Ovarian Cancer-Secreted Exosomal miR-222-3p Induces Polarization of Tumor-Associated Macrophages. Oncotarget (2016) 7(28):43076–87. doi: 10.18632/oncotarget.9246 PMC5190009 27172798

[47] Ding G Zhou L Qian Y Fu M Chen J Chen J. Pancreatic Cancer-Derived Exosomes Transfer miRNAs to Dendritic Cells and Inhibit RFXAP Expression *via* miR-212-3p. Oncotarget (2015) 6(30):29877–88. doi: 10.18632/oncotarget.4924 PMC4745769 26337469

[48] Richards KE Zeleniak AE Fishel ML Wu J Littlepage LE Hill R. Cancer-Associated Fibroblast Exosomes Regulate Survival and Proliferation of Pancreatic Cancer Cells. Oncogene (2017) 36(13):1770–8. doi: 10.1038/onc.2016.353 PMC5366272 27669441

[49] Momen-Heravi F Balaj L Alian S Trachtenberg AJ Hochberg FH Skog J. Impact of Biofluid Viscosity on Size and Sedimentation Efficiency of the Isolated Microvesicles. Front Physiol (2012) 3:162. doi: 10.3389/fphys.2012.00162 22661955 PMC3362089

[50] Witwer KW Buzas EI Bemis LT Bora A Lasser C Lotvall J. Standardization of Sample Collection, Isolation and Analysis Methods in Extracellular Vesicle Research. J Extracell Vesicles (2013) 2(1):20360. doi: 10.3402/jev.v2i0.20360 PMC3760646 24009894

[51] Jung HH Kim JY Lim JE Im YH. Cytokine Profiling in Serum-Derived Exosomes Isolated by Different Methods. Sci Rep (2020) 10(1):14069. doi: 10.1038/s41598-020-70584-z 32826923 PMC7442638

[52] Taylor DD Shah S. Methods of Isolating Extracellular Vesicles Impact Down-Stream Analyses of Their Cargoes. Methods (2015) 87:3–10. doi: 10.1016/j.ymeth.2015.02.019 25766927

[53] Nakai W Yoshida T Diez D Miyatake Y Nishibu T Imawaka N. A Novel Affinity-Based Method for the Isolation of Highly Purified Extracellular Vesicles. Sci Rep (2016) 6:33935. doi: 10.1038/srep33935 27659060 PMC5034288

[54] Ning B Huang Z Youngquist BM Scott JW Niu A Bojanowski CM. Liposome-Mediated Detection of SARS-CoV-2 RNA-Positive Extracellular Vesicles in Plasma. Nat Nanotechnol (2021) 16(9):1039–44. doi: 10.1038/s41565-021-00939-8 PMC8440422 34294909

[55] Li P Kaslan M Lee SH Yao J Gao Z. Progress in Exosome Isolation Techniques. Theranostics (2017) 7(3):789–804. doi: 10.7150/thno.18133 28255367 PMC5327650

[56] Baranyai T Herczeg K Onodi Z Voszka I Modos K Marton N. Isolation of Exosomes From Blood Plasma: Qualitative and Quantitative Comparison of Ultracentrifugation and Size Exclusion Chromatography Methods. PloS One (2015) 10(12):e0145686. doi: 10.1371/journal.pone.0145686 26690353 PMC4686892

[57] Hong P Koza S Bouvier ES. Size-Exclusion Chromatography for the Analysis of Protein Biotherapeutics and Their Aggregates. J Liq Chromatogr Relat Technol (2012) 35(20):2923–50. doi: 10.1080/10826076.2012.743724 PMC3556795 23378719

[58] Clayton A Court J Navabi H Adams M Mason MD Hobot JA. Analysis of Antigen Presenting Cell Derived Exosomes, Based on Immuno-Magnetic Isolation and Flow Cytometry. J Immunol Methods (2001) 247(1-2):163–74. doi: 10.1016/s0022-1759(00)00321-5 11150547

[59] Zhang K Yue Y Wu S Liu W Shi J Zhang Z. Rapid Capture and Nondestructive Release of Extracellular Vesicles Using Aptamer-Based Magnetic Isolation. ACS Sensors (2019) 4(5):1245–51. doi: 10.1021/acssensors.9b00060 30915846

[60] Lobb RJ Becker M Wen SW Wong CS Wiegmans AP Leimgruber A. Optimized Exosome Isolation Protocol for Cell Culture Supernatant and Human Plasma. J Extracell Vesicles (2015) 4:27031. doi: 10.3402/jev.v4.27031 26194179 PMC4507751

[61] Nordin JZ Lee Y Vader P Mager I Johansson HJ Heusermann W. Ultrafiltration With Size-Exclusion Liquid Chromatography for High Yield Isolation of Extracellular Vesicles Preserving Intact Biophysical and Functional Properties. Nanomedicine (2015) 11(4):879–83. doi: 10.1016/j.nano.2015.01.003 25659648

[62] Shurtleff MJ Temoche-Diaz MM Schekman R. Extracellular Vesicles and Cancer: Caveat Lector. Annu Rev Cancer Biol (2018) 2(1):395–411. doi: 10.1146/annurev-cancerbio-030617-050519

[63] Jeppesen DK Fenix AM Franklin JL Higginbotham JN Zhang Q Zimmerman LJ. Reassessment of Exosome Composition. Cell (2019) 177(2):428–445 e18. doi: 10.1016/j.cell.2019.02.029 30951670 PMC6664447

[64] van der Pol E Hoekstra AG Sturk A Otto C van Leeuwen TG Nieuwland R. Optical and Non-Optical Methods for Detection and Characterization of Microparticles and Exosomes. J Thromb Haemost (2010) 8(12):2596–607. doi: 10.1111/j.1538-7836.2010.04074.x 20880256

[65] Dragovic RA Gardiner C Brooks AS Tannetta DS Ferguson DJ Hole P. Sizing and Phenotyping of Cellular Vesicles Using Nanoparticle Tracking Analysis. Nanomedicine (2011) 7(6):780–8. doi: 10.1016/j.nano.2011.04.003 PMC3280380 21601655

[66] Coumans FAW Gool EL Nieuwland R. Bulk Immunoassays for Analysis of Extracellular Vesicles. Platelets (2017) 28(3):242–8. doi: 10.1080/09537104.2016.1265926 28102735

[67] Karimi N Cvjetkovic A Jang SC Crescitelli R Hosseinpour Feizi MA Nieuwland R. Detailed Analysis of the Plasma Extracellular Vesicle Proteome After Separation From Lipoproteins. Cell Mol Life Sci (2018) 75(15):2873–86. doi: 10.1007/s00018-018-2773-4 PMC6021463 29441425

[68] Feng Y Zhong X Tang T-T Wang C Wang L-T Li Z-L. Rab27a Dependent Exosome Releasing Participated in Albumin Handling as a Coordinated Approach to Lysosome in Kidney Disease. Cell Death Dis (2020) 11(7):513. doi: 10.1038/s41419-020-2709-4 32641688 PMC7343869

[69] Li C Zhou Y Liu J Su X Qin H Huang S. Potential Markers From Serum-Purified Exosomes for Detecting Oral Squamous Cell Carcinoma Metastasis. Cancer Epidemiol Biomarkers Prev (2019) 28(10):1668–81. doi: 10.1158/1055-9965.Epi-18-1122 31350263

[70] Lacroix R Robert S Poncelet P Dignat-George F. Overcoming Limitations of Microparticle Measurement by Flow Cytometry. Semin Thromb Hemost (2010) 36(8):807–18. doi: 10.1055/s-0030-1267034 21049381

[71] Kowal J Arras G Colombo M Jouve M Morath JP Primdal-Bengtson B. Proteomic Comparison Defines Novel Markers to Characterize Heterogeneous Populations of Extracellular Vesicle Subtypes. Proc Natl Acad Sci USA (2016) 113(8):E968–77. doi: 10.1073/pnas.1521230113 PMC4776515 26858453

[72] Contreras-Naranjo JC Wu HJ Ugaz VM. Microfluidics for Exosome Isolation and Analysis: Enabling Liquid Biopsy for Personalized Medicine. Lab Chip (2017) 17(21):3558–77. doi: 10.1039/c7lc00592j PMC5656537 28832692

[73] Ueda K Ishikawa N Tatsuguchi A Saichi N Fujii R Nakagawa H. Antibody-Coupled Monolithic Silica Microtips for Highthroughput Molecular Profiling of Circulating Exosomes. Sci Rep (2014) 4, 6232. doi: 10.1038/srep06232 25167841 PMC4148700

[74] Sandfeld-Paulsen B Jakobsen KR Baek R Folkersen BH Rasmussen TR Meldgaard P. Exosomal Proteins as Diagnostic Biomarkers in Lung Cancer. J Thorac Oncol (2016) 11(10):1701–10. doi: 10.1016/j.jtho.2016.05.034 27343445

[75] Wang X Zhong W Bu J Li Y Li R Nie R. Exosomal Protein CD82 as a Diagnostic Biomarker for Precision Medicine for Breast Cancer. Mol Carcinog (2019) 58(5):674–85. doi: 10.1002/mc.22960 30604894

[76] Yoshioka Y Kosaka N Konishi Y Ohta H Okamoto H Sonoda H. Ultra-Sensitive Liquid Biopsy of Circulating Extracellular Vesicles Using ExoScreen. Nat Commun (2014) 5, 3591. doi: 10.1038/ncomms4591 24710016 PMC3988821

[77] Madhavan B Yue S Galli U Rana S Gross W Muller M. Combined Evaluation of a Panel of Protein and miRNA Serum-Exosome Biomarkers for Pancreatic Cancer Diagnosis Increases Sensitivity and Specificity. Int J Cancer (2015) 136(11):2616–27. doi: 10.1002/ijc.29324 25388097

[78] Krishn SR Singh A Bowler N Duffy AN Friedman A Fedele C. Prostate Cancer Sheds the Alphavbeta3 Integrin *In Vivo* Through Exosomes. Matrix Biol (2019) 77:41–57. doi: 10.1016/j.matbio.2018.08.004 30098419 PMC6541230

[79] Soekmadji C Riches JD Russell PJ Ruelcke JE McPherson S Wang C. Modulation of Paracrine Signaling by CD9 Positive Small Extracellular Vesicles Mediates Cellular Growth of Androgen Deprived Prostate Cancer. Oncotarget (2017) 8(32):52237–55. doi: 10.18632/oncotarget.11111 PMC5581025 28881726

[80] Kato T Mizutani K Kameyama K Kawakami K Fujita Y Nakane K. Serum Exosomal P-Glycoprotein Is a Potential Marker to Diagnose Docetaxel Resistance and Select a Taxoid for Patients With Prostate Cancer. Urol Oncol (2015) 33(9):385 e15–20. doi: 10.1016/j.urolonc.2015.04.019 26027763

[81] Logozzi M De Milito A Lugini L Borghi M Calabro L Spada M. High Levels of Exosomes Expressing CD63 and Caveolin-1 in Plasma of Melanoma Patients. PloS One (2009) 4(4):e5219. doi: 10.1371/journal.pone.0005219 19381331 PMC2667632

[82] Rodriguez Zorrilla S Perez-Sayans M Fais S Logozzi M Gallas Torreira M Garcia Garcia A. A Pilot Clinical Study on the Prognostic Relevance of Plasmatic Exosomes Levels in Oral Squamous Cell Carcinoma Patients. Cancers (Basel) (2019) 11(3):429. doi: 10.3390/cancers11030429 PMC6468603 30917536

[83] Chanteloup G Cordonnier M Isambert N Bertaut A Hervieu A Hennequin A. Monitoring HSP70 Exosomes in Cancer Patients' Follow Up: A Clinical Prospective Pilot Study. J Extracell Vesicles (2020) 9(1):1766192. doi: 10.1080/20013078.2020.1766192 32595915 PMC7301715

[84] An T Qin S Sun D Huang Y Hu Y Li S. Unique Protein Profiles of Extracellular Vesicles as Diagnostic Biomarkers for Early and Advanced Non-Small Cell Lung Cancer. Proteomics (2019) 19(12):e1800160. doi: 10.1002/pmic.201800160 30950185

[85] Ding XQ Wang ZY Xia D Wang RX Pan XR Tong JH. Proteomic Profiling of Serum Exosomes From Patients With Metastatic Gastric Cancer. Front Oncol (2020) 10:1113. doi: 10.3389/fonc.2020.01113 32754443 PMC7367030

[86] Chen Y Xie Y Xu L Zhan S Xiao Y Gao Y. Protein Content and Functional Characteristics of Serum-Purified Exosomes From Patients With Colorectal Cancer Revealed by Quantitative Proteomics. Int J Cancer (2017) 140(4):900–13. doi: 10.1002/ijc.30496 27813080

[87] Ishizuya Y Uemura M Narumi R Tomiyama E Koh Y Matsushita M. The Role of Actinin-4 (ACTN4) in Exosomes as a Potential Novel Therapeutic Target in Castration-Resistant Prostate Cancer. Biochem Biophys Res Commun (2020) 523(3):588–94. doi: 10.1016/j.bbrc.2019.12.084 31941606

[88] Jin H Liu P Wu Y Meng X Wu M Han J. Exosomal Zinc Transporter ZIP4 Promotes Cancer Growth and Is a Novel Diagnostic Biomarker for Pancreatic Cancer. Cancer Sci (2018) 109(9):2946–56. doi: 10.1111/cas.13737 PMC6125444 30007115

[89] Herrero C Abal M Muinelo-Romay L. Circulating Extracellular Vesicles in Gynecological Tumors: Realities and Challenges. Front Oncol (2020) 10:565666. doi: 10.3389/fonc.2020.565666 33178595 PMC7591787

[90] Wyciszkiewicz A Kalinowska-Łyszczarz A Nowakowski B Kaźmierczak K Osztynowicz K Michalak S. Expression of Small Heat Shock Proteins in Exosomes From Patients With Gynecologic Cancers. Sci Rep (2019) 9(1), 9817. doi: 10.1038/s41598-019-46221-9 31285463 PMC6614356

[91] Shao H Chung J Balaj L Charest A Bigner DD Carter BS. Protein Typing of Circulating Microvesicles Allows Real-Time Monitoring of Glioblastoma Therapy. Nat Med (2012) 18(12):1835–40. doi: 10.1038/nm.2994 PMC3518564 23142818

[92] Yamashita T Kamada H Kanasaki S Maeda Y Nagano K Abe Y. Epidermal Growth Factor Receptor Localized to Exosome Membranes as a Possible Biomarker for Lung Cancer Diagnosis. Pharmazie (2013) 68(12):969–73. doi: 10.1691/ph.2013.3599 24400444

[93] Arbelaiz A Azkargorta M Krawczyk M Santos-Laso A Lapitz A Perugorria MJ. Serum Extracellular Vesicles Contain Protein Biomarkers for Primary Sclerosing Cholangitis and Cholangiocarcinoma. Hepatology (2017) 66(4):1125–43. doi: 10.1002/hep.29291 28555885

[94] Li J Chen Y Guo X Zhou L Jia Z Peng Z. GPC1 Exosome and its Regulatory miRNAs Are Specific Markers for the Detection and Target Therapy of Colorectal Cancer. J Cell Mol Med (2017) 21(5):838–47. doi: 10.1111/jcmm.12941 PMC5387162 28233416

[95] Li J Li B Ren C Chen Y Guo X Zhou L. The Clinical Significance of Circulating GPC1 Positive Exosomes and Its Regulative miRNAs in Colon Cancer Patients. Oncotarget (2017) 8(60):101189–202. doi: 10.18632/oncotarget.20516 PMC5731866 29254156

[96] Lux A Kahlert C Grutzmann R Pilarsky C. C-Met and PD-L1 on Circulating Exosomes as Diagnostic and Prognostic Markers for Pancreatic Cancer. Int J Mol Sci (2019) 20(13):3305. doi: 10.3390/ijms20133305 PMC6651266 31284422

[97] Melo SA Luecke LB Kahlert C Fernandez AF Gammon ST Kaye J. Glypican-1 Identifies Cancer Exosomes and Detects Early Pancreatic Cancer. Nature (2015) 523(7559):177–82. doi: 10.1038/nature14581 PMC4825698 26106858

[98] Frampton AE Prado MM Lopez-Jimenez E Fajardo-Puerta AB Jawad ZAR Lawton P. Glypican-1 Is Enriched in Circulating-Exosomes in Pancreatic Cancer and Correlates With Tumor Burden. Oncotarget (2018) 9(27):19006–13. doi: 10.18632/oncotarget.24873 PMC5922373 29721179

[99] Xiao D Dong Z Zhen L Xia G Huang X Wang T. Combined Exosomal GPC1, CD82, and Serum CA19-9 as Multiplex Targets: A Specific, Sensitive, and Reproducible Detection Panel for the Diagnosis of Pancreatic Cancer. Mol Cancer Res (2020) 18(2):300–10. doi: 10.1158/1541-7786.MCR-19-0588 31662449

[100] Buscail E Chauvet A Quincy P Degrandi O Buscail C Lamrissi I. CD63-GPC1-Positive Exosomes Coupled With CA19-9 Offer Good Diagnostic Potential for Resectable Pancreatic Ductal Adenocarcinoma. Transl Oncol (2019) 12(11):1395–403. doi: 10.1016/j.tranon.2019.07.009 PMC6699195 31400579

[101] Zhao Z Yang Y Zeng Y He M. A Microfluidic ExoSearch Chip for Multiplexed Exosome Detection Towards Blood-Based Ovarian Cancer Diagnosis. Lab Chip (2016) 16(3):489–96. doi: 10.1039/c5lc01117e PMC4729647 26645590

[102] Chen G Huang AC Zhang W Zhang G Wu M Xu W. Exosomal PD-L1 Contributes to Immunosuppression and Is Associated With Anti-PD-1 Response. Nature (2018) 560(7718):382–6. doi: 10.1038/s41586-018-0392-8 PMC6095740 30089911

[103] Theodoraki MN Yerneni SS Hoffmann TK Gooding WE Whiteside TL. Clinical Significance of PD-L1(+) Exosomes in Plasma of Head and Neck Cancer Patients. Clin Cancer Res (2018) 24(4):896–905. doi: 10.1158/1078-0432.CCR-17-2664 29233903 PMC6126905

[104] Erozenci LA Böttger F Bijnsdorp IV Jimenez CR. Urinary Exosomal Proteins as (Pan-)Cancer Biomarkers: Insights From the Proteome. FEBS Lett (2019) 593(13):1580–97. doi: 10.1002/1873-3468.13487 31198995

[105] Chen CL Lai YF Tang P Chien KY Yu JS Tsai CH. Comparative and Targeted Proteomic Analyses of Urinary Microparticles From Bladder Cancer and Hernia Patients. J Proteome Res (2012) 11(12):5611–29. doi: 10.1021/pr3008732 23082778

[106] Sakaue T Koga H Iwamoto H Nakamura T Ikezono Y Abe M. Glycosylation of Ascites-Derived Exosomal CD133: A Potential Prognostic Biomarker in Patients With Advanced Pancreatic Cancer. Med Mol Morphol (2019) 52(4):198–208. doi: 10.1007/s00795-019-00218-5 30805710

[107] Pisitkun T Shen RF Knepper MA. Identification and Proteomic Profiling of Exosomes in Human Urine. Proc Natl Acad Sci USA (2004) 101(36):13368–73. doi: 10.1073/pnas.0403453101 PMC516573 15326289

[108] Jalalian SH Ramezani M Jalalian SA Abnous K Taghdisi SM. Exosomes, New Biomarkers in Early Cancer Detection. Anal Biochem (2019) 571:1–13. doi: 10.1016/j.ab.2019.02.013 30776327

[109] He M Crow J Roth M Zeng Y Godwin AK. Integrated Immunoisolation and Protein Analysis of Circulating Exosomes Using Microfluidic Technology. Lab Chip (2014) 14(19):3773–80. doi: 10.1039/c4lc00662c PMC4161194 25099143

[110] Jakobsen KR Paulsen BS Baek R Varming K Sorensen BS Jorgensen MM. Exosomal Proteins as Potential Diagnostic Markers in Advanced Non-Small Cell Lung Carcinoma. J Extracell Vesicles (2015) 4:26659. doi: 10.3402/jev.v4.26659 25735706 PMC4348413

[111] Jorgensen M Baek R Pedersen S Sondergaard EK Kristensen SR Varming K. Extracellular Vesicle (EV) Array: Microarray Capturing of Exosomes and Other Extracellular Vesicles for Multiplexed Phenotyping. J Extracell Vesicles (2013) 2:20920. doi: 10.3402/jev.v2i0.20920 PMC3760630 24009888

[112] Malla RR Pandrangi S Kumari S Gavara MM Badana AK. Exosomal Tetraspanins as Regulators of Cancer Progression and Metastasis and Novel Diagnostic Markers. Asia-Pacific J Clin Oncol (2018) 14(6):383–91. doi: 10.1111/ajco.12869 29575602

[113] Uhlen M Bjorling E Agaton C Szigyarto CA Amini B Andersen E. A Human Protein Atlas for Normal and Cancer Tissues Based on Antibody Proteomics. Mol Cell Proteomics (2005) 4(12):1920–32. doi: 10.1074/mcp.M500279-MCP200 16127175

[114] Yurchenko V Constant S Bukrinsky M. Dealing With the Family: CD147 Interactions With Cyclophilins. Immunology (2006) 117(3):301–9. doi: 10.1111/j.1365-2567.2005.02316.x PMC1782239 16476049

[115] Li Y Yu S Li L Chen J Quan M Li Q. KLF4-Mediated Upregulation of CD9 and CD81 Suppresses Hepatocellular Carcinoma Development *via* JNK Signaling. Cell Death Dis (2020) 11(4):299. doi: 10.1038/s41419-020-2479-z 32350244 PMC7190708

[116] Khushman M Prodduturvar P Mneimneh W Zotto VD Akbar S Grimm L. The Impact of Neoadjuvant Concurrent Chemoradiation on Exosomal Markers (CD63 and CD9) Expression and Their Prognostic Significance in Patients With Rectal Adenocarcinoma. Oncotarget (2021) 12(15):1490–8. doi: 10.18632/oncotarget.28025 PMC8310674 34316329

[117] Oh KB Stanton MJ West WW Todd GL Wagner KU. Tsg101 is Upregulated in a Subset of Invasive Human Breast Cancers and Its Targeted Overexpression in Transgenic Mice Reveals Weak Oncogenic Properties for Mammary Cancer Initiation. Oncogene (2007) 26(40):5950–9. doi: 10.1038/sj.onc.1210401 17369844

[118] Yang J Zhang Y Gao X Yuan Y Zhao J Zhou S. Plasma-Derived Exosomal ALIX as a Novel Biomarker for Diagnosis and Classification of Pancreatic Cancer. Front Oncol (2021) 11:628346. doi: 10.3389/fonc.2021.628346 34026608 PMC8131866

[119] Albakova Z Siam MKS Sacitharan PK Ziganshin RH Ryazantsev DY Sapozhnikov AM. Extracellular Heat Shock Proteins and Cancer: New Perspectives. Trans Oncol (2021) 14(2):100995. doi: 10.1016/j.tranon.2020.100995 PMC7749402 33338880

[120] Gheytanchi E Saeednejad Zanjani L Ghods R Abolhasani M Shahin M Vafaei S. High Expression of Tumor Susceptibility Gene 101 (TSG101) Is Associated With More Aggressive Behavior in Colorectal Carcinoma. J Cancer Res Clin Oncol (2021) 147(6):1631–46. doi: 10.1007/s00432-021-03561-2 PMC11801982 33616717

[121] Albakova Z Armeev GA Kanevskiy LM Kovalenko EI Sapozhnikov AM. HSP70 Multi-Functionality in Cancer. Cells (2020) 9(3):587. doi: 10.3390/cells9030587 PMC7140411 32121660

[122] Bausero MA Gastpar R Multhoff G Asea A. Alternative Mechanism by Which IFN-γ Enhances Tumor Recognition: Active Release of Heat Shock Protein 72. J Immunol (2005) 175(5):2900–12. doi: 10.4049/jimmunol.175.5.2900 PMC1762097 16116176

[123] Jiang C Cao S Li N Jiang L Sun T. PD-1 and PD-L1 Correlated Gene Expression Profiles and Their Association With Clinical Outcomes of Breast Cancer. Cancer Cell Int (2019) 19:233. doi: 10.1186/s12935-019-0955-2 31516390 PMC6734479

[124] Li N Gao W Zhang Y-F Ho M. Glypicans as Cancer Therapeutic Targets. Trends Cancer (2018) 4(11):741–54. doi: 10.1016/j.trecan.2018.09.004 PMC6209326 30352677

[125] Lund ME Campbell DH Walsh BJ. The Role of Glypican-1 in the Tumour Microenvironment. Adv Exp Med Biol (2020) 1245:163–76. doi: 10.1007/978-3-030-40146-7_8 32266658

[126] Yang KS Im H Hong S Pergolini I Del Castillo AF Wang R. Multiparametric Plasma EV Profiling Facilitates Diagnosis of Pancreatic Malignancy. Sci Transl Med (2017) 9(391):eaal3226. doi: 10.1126/scitranslmed.aal3226 28539469 PMC5846089

[127] Soltesz B Lukacs J Szilagyi E Marton E Szilagyi Bonizs M Penyige A. Expression of CD24 in Plasma, Exosome and Ovarian Tissue Samples of Serous Ovarian Cancer Patients. J Biotechnol (2019) 298:16–20. doi: 10.1016/j.jbiotec.2019.03.018 30959137

[128] Khan S Bennit HF Turay D Perez M Mirshahidi S Yuan Y. Early Diagnostic Value of Survivin and Its Alternative Splice Variants in Breast Cancer. BMC Cancer (2014) 14:176. doi: 10.1186/1471-2407-14-176 24620748 PMC3995700

[129] Wang H Lu Z Zhao X. Tumorigenesis, Diagnosis, and Therapeutic Potential of Exosomes in Liver Cancer. J Hematol Oncol (2019) 12(1):133. doi: 10.1186/s13045-019-0806-6 31815633 PMC6902437

[130] Zhou H Yuen PS Pisitkun T Gonzales PA Yasuda H Dear JW. Collection, Storage, Preservation, and Normalization of Human Urinary Exosomes for Biomarker Discovery. Kidney Int (2006) 69(8):1471–6. doi: 10.1038/sj.ki.5000273 PMC2276656 16501490

[131] Wang Y-T Shi T Srivastava S Kagan J Liu T Rodland KD. Proteomic Analysis of Exosomes for Discovery of Protein Biomarkers for Prostate and Bladder Cancer. Cancers (2020) 12(9), 2335. doi: 10.3390/cancers12092335 PMC7564640 32825017

[132] Georgantzoglou N Pergaris A Masaoutis C Theocharis S. Extracellular Vesicles as Biomarkers Carriers in Bladder Cancer: Diagnosis, Surveillance, and Treatment. Int J Mol Sci (2021) 22(5), 2744. doi: 10.3390/ijms22052744 33803085 PMC7963171

[133] Li Y Zhang Y Qiu F Qiu Z. Proteomic Identification of Exosomal LRG1: A Potential Urinary Biomarker for Detecting NSCLC. Electrophoresis (2011) 32(15):1976–83. doi: 10.1002/elps.201000598 21557262

[134] Mizenko RR Brostoff T Rojalin T Koster HJ Swindell HS Leiserowitz GS. Tetraspanins Are Unevenly Distributed Across Single Extracellular Vesicles and Bias Sensitivity to Multiplexed Cancer Biomarkers. J Nanobiotech (2021) 19(1):250. doi: 10.1186/s12951-021-00987-1 PMC8379740 34419056

[135] Lin S Yu Z Chen D Wang Z Miao J Li Q. Progress in Microfluidics-Based Exosome Separation and Detection Technologies for Diagnostic Applications. Small (2020) 16(9):e1903916. doi: 10.1002/smll.201903916 31663295

[136] Zhang W Tian Z Yang S Rich J Zhao S Klingeborn M. Electrochemical Micro-Aptasensors for Exosome Detection Based on Hybridization Chain Reaction Amplification. Microsystems Nanoeng (2021) 7(1):63. doi: 10.1038/s41378-021-00293-8 PMC8433316 34567775

[137] Wang L Zeng L Wang Y Chen T Chen W Chen G. Electrochemical Aptasensor Based on Multidirectional Hybridization Chain Reaction for Detection of Tumorous Exosomes. Sensors Actuators B: Chem (2021) 332:129471. doi: 10.1016/j.snb.2021.129471

[138] Palviainen M Saraswat M Varga Z Kitka D Neuvonen M Puhka M. Extracellular Vesicles From Human Plasma and Serum Are Carriers of Extravesicular Cargo-Implications for Biomarker Discovery. PloS One (2020) 15(8):e0236439. doi: 10.1371/journal.pone.0236439 32813744 PMC7446890

[139] Dal Collo G Adamo A Gatti A Tamellini E Bazzoni R Takam Kamga P. Functional Dosing of Mesenchymal Stromal Cell-Derived Extracellular Vesicles for the Prevention of Acute Graft-Versus-Host-Disease. Stem Cells (2020) 38(5):698–711. doi: 10.1002/stem.3160 32064745

[140] Lai P Chen X Guo L Wang Y Liu X Liu Y. A Potent Immunomodulatory Role of Exosomes Derived From Mesenchymal Stromal Cells in Preventing cGVHD. J Hematol Oncol (2018) 11(1):135. doi: 10.1186/s13045-018-0680-7 30526632 PMC6286548

[141] Kordelas L Rebmann V Ludwig AK Radtke S Ruesing J Doeppner TR. MSC-Derived Exosomes: A Novel Tool to Treat Therapy-Refractory Graft-Versus-Host Disease. Leukemia (2014) 28(4):970–3. doi: 10.1038/leu.2014.41 24445866

[142] Sengupta V Sengupta S Lazo A Woods P Nolan A Bremer N. Exosomes Derived From Bone Marrow Mesenchymal Stem Cells as Treatment for Severe COVID-19. Stem Cells Dev (2020) 29(12):747–54. doi: 10.1089/scd.2020.0080 PMC7310206 32380908

